# Supplementary material for: Nucleobase synthesis in interstellar ices
Source: Nat Commun. 2019 Sep 27;10:4413. doi: 10.1038/s41467-019-12404-1 (PMC6764953; doi:10.1038/s41467-019-12404-1)
Supplement: Supplementary file 1 — Supplementary Information [file 41467_2019_12404_MOESM1_ESM.docx]

Supplementary Information for

**Nucleobase synthesis in interstellar ices**

Yasuhiro Oba^1*^, Yoshinori Takano^2^, Hiroshi Naraoka^3,4^, Naoki Watanabe^1^,

and Akira Kouchi^1^

**Affiliations:**

^1^Institute of Low Temperature Science (ILTS), Hokkaido University, N19W8, Kita-ku, Sapporo, Hokkaido 060-0189 Japan.

^2^†Department of Biogeochemistry, Japan Agency for Marine-Earth Science and Technology (JAMSTEC), 2-15 Natsushima, Yokosuka, Kanagawa 237-0061 Japan.

^3^Department of Earth and Planetary Sciences, Kyushu University, 744 Motooka, Nishi-ku, Fukuoka, Fukuoka 819-0395 Japan.

^4^Research Center for Planetary Trace Organic Compounds, Kyushu University, 744 Motooka, Nishi-ku, Fukuoka, Fukuoka 819-0395 Japan

Contents:

•Supplementary Notes 1–2

•Supplementary Figures 1–35

•Supplementary Tables 1–2

•Supplementary References

Supplementary Note 1: Structural isomers of nucleobases in the sample.

A number of peaks appeared in each mass chromatogram and these were due to compounds other than nucleobases (e.g., Figs. 2 and 3). In the case of uracil, for example, the intensity of the peak due to this nucleobase is much smaller than those of most other peaks present in the same chromatogram. The same thing is true for other nucleobases present in the sample (Supplementary Figures 1−6). Based on the mass resolution (*Δm*/*m* = 140,000 at a mass-to-charge ratio of 200) and precision (< 3 ppm) of the spectral data collected in the present analysis, the peaks observed in the spectrum reported in Fig. 2 should be due to molecules characterised by a C_4_H_4_N_2_O_2_ molecular formula (i.e. structural isomers of uracil). Under that analytical condition, if mass analysis is not accompanied by the performance of a chromatographic separation, the abundance of a target molecule (a nucleobase, in the present case) is very likely to be overestimated in a complex mixture, due to the possible presence in the analyte sample of the target molecule’s structural isomers. Hence, results from the present study strongly suggest that the identification of molecules in complex mixtures should not rely on mass analysis alone but on a combination of multiple analytical approaches. Moreover, even if the composition of a sample has been investigated by chromatographic separation in combination with mass analysis, the implementation of additional analytical approaches under different conditions is highly desirable to further confirm the presence of target molecules. In that sense, the detection of nucleobases obtained by the co-injection of the analyte samples and standard reagents in the two different analytical conditions described strongly supports our conclusion that these species are actually present in the organic residues produced by a mixture of simple molecules.

**Supplementary Note 2:** **Possible roles for carbon monoxide in nucleobase formation**.

Carbon monoxide (CO) plays an important role in the formation of various molecules, such as methanol (CH_3_OH), formaldehyde (H_2_CO) and carbon dioxide (CO_2_) via surface reactions^1^. Wang and Bowie^2^ proposed a pathway for thymine formation that begins with the reaction of isocyanic acid (HNCO) with propanal (CH_3_CH_2_CHO), two compounds that have been detected in the interstellar medium^3,4^. Since HNCO can form in a mixture of solid CO and nitrogen-containing species (e.g. NH_3_ and NO) exposed to UV photons^5^, we expect that this acid can form in the interstellar ice analogues utilised in the present study. In addition, formamide (HCONH_2_) has been proposed to be a key precursor for the abiotic synthesis of nucleobases under warm conditions^6–10^. Nucleobase formation was reported to occur when liquid or solid HCONH_2_ was exposed to high-energy particles at temperatures above 77 K^6,7,9,10^. Furthermore, since HCONH_2_ can be produced in a binary mixture of CO and NH_3_ after exposure of the said mixture to energetic particles at temperatures as low as ~10 K^11,12^, HCONH_2_ formation is highly expected to occur under the present experimental conditions. On the other hand, given that the syntheses of HNCO and HCONH_2_ through the CO-derived pathways are suppressed at 77 K where CO cannot adsorb on the substrate, the suppression of nucleobase formation is to be expected at this temperature. In fact, the pioneering experimental result that nucleobases were not detected in a sample exposed to UV photons at 77 K^13^ is not in contradiction with the mentioned expectation.

**Supplementary Figures**


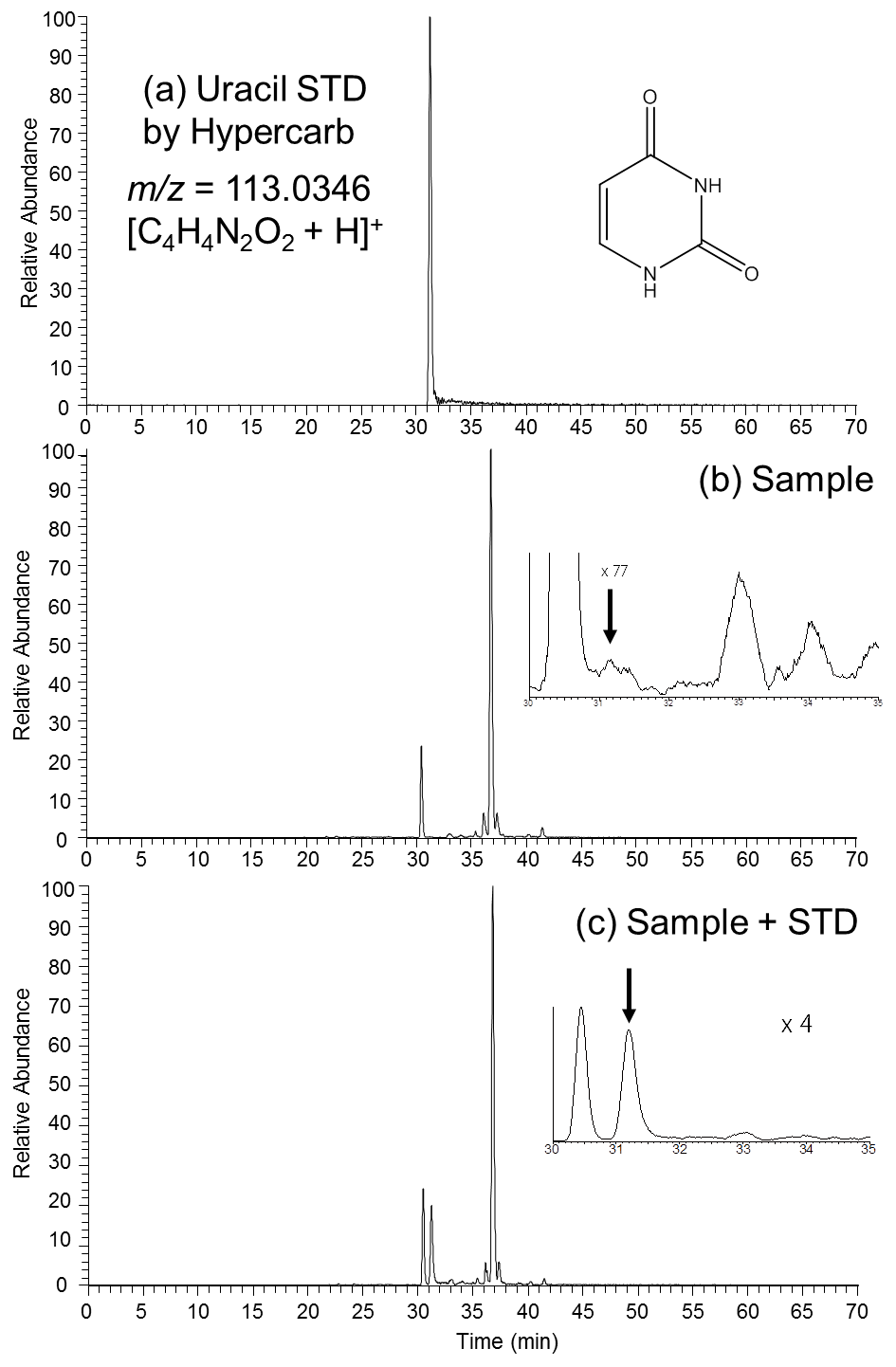


Supplementary Figure 1. Identification of uracil in the organic residues. Mass chromatograms of (a) the uracil standard, (b) the analyte sample, and (c) the co-injected mixture of the uracil standard and the analyte sample at the mass-to-charge ratio (*m/z*) of 113.0346. A Hypercarb separation column was used on the analysis by HPLC. The solid arrow indicates uracil present in the samples. Inset shows an enlarged spectrum at 30 and 35 min.


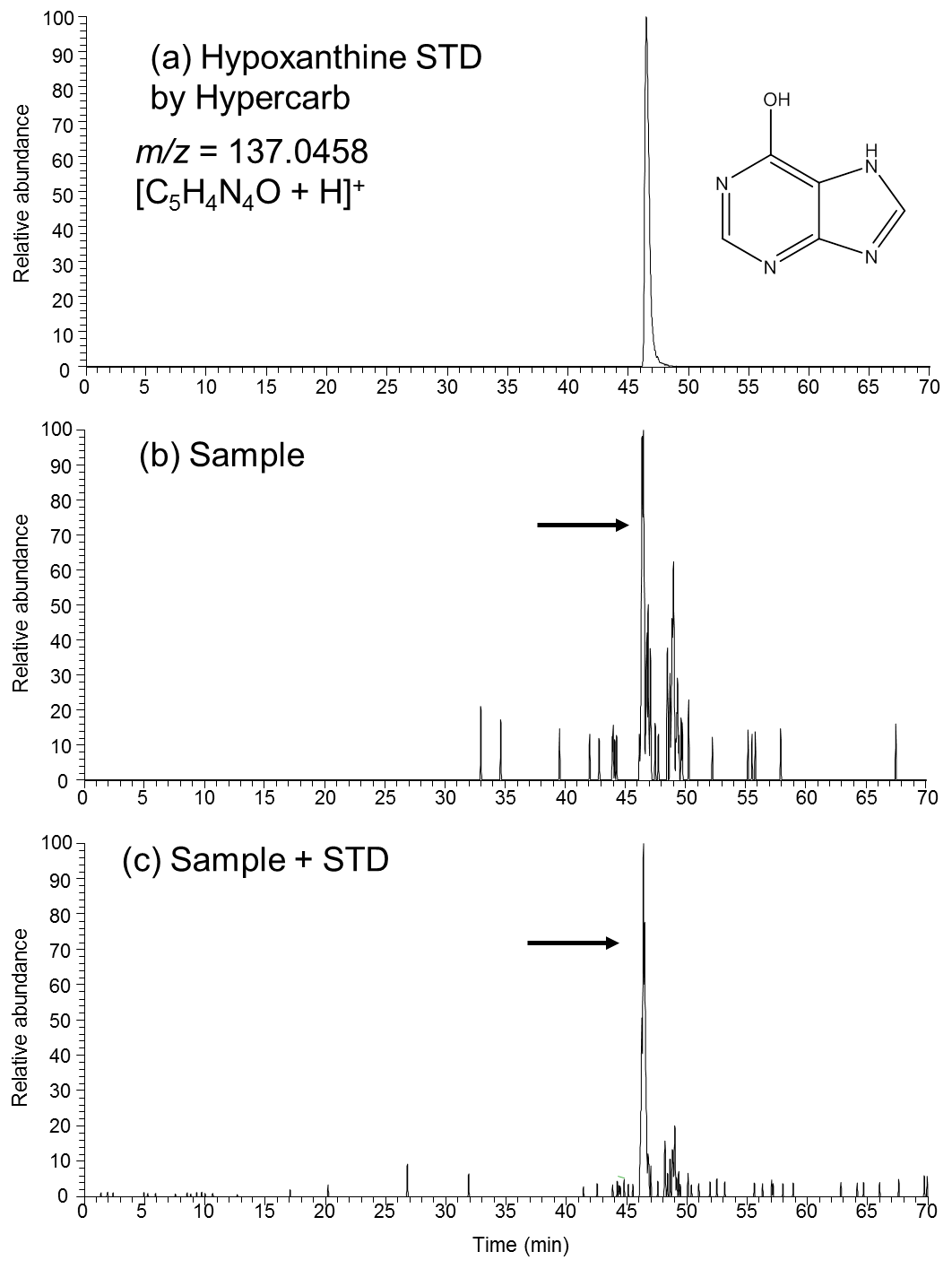


Supplementary Figure 2. Identification of hypoxanthine in the organic residues. Mass chromatograms of (a) the hypoxanthine standard, (b) the analyte sample and (c) the co-injected mixture of hypoxanthine standard and analyte sample at the *m/z* of 137.0458. A Hypercarb^TM^ separation column was used for the HPLC/HRMS analysis. The solid arrow indicates the peak due to hypoxanthine.


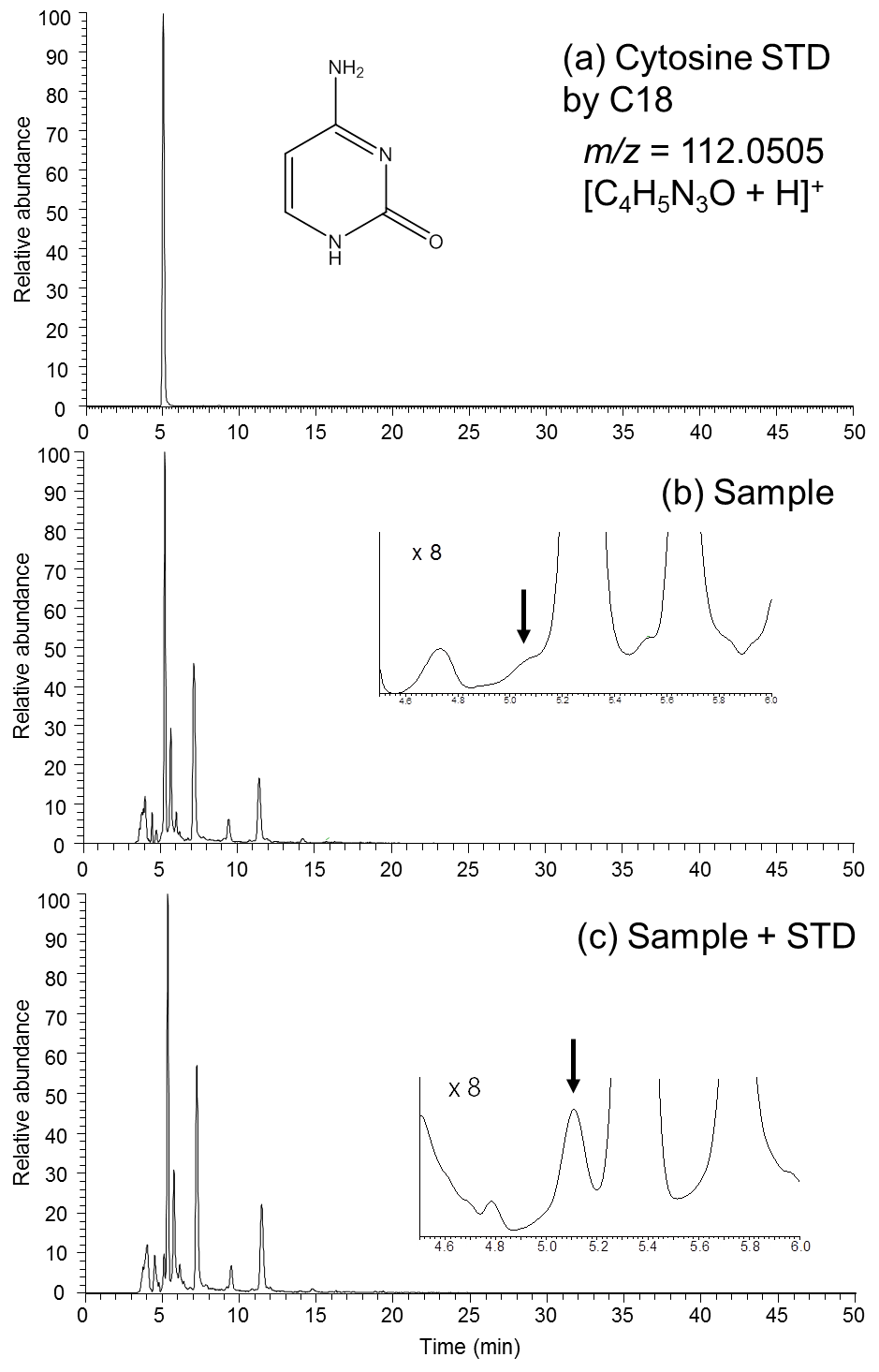


Supplementary Figure 3A. Identification of cytosine in the organic residues. Mass chromatograms of (a) the cytosine standard, (b) the analyte sample and (c) the co-injected mixture of cytosine standard and analyte sample at the *m/z* of 112.0505. A C18 separation column was used for the HPLC/HRMS analysis. The solid arrow indicates cytosine. The inset consists of a blow-up of the section of the spectrum between 4.5 and 6.0 min.


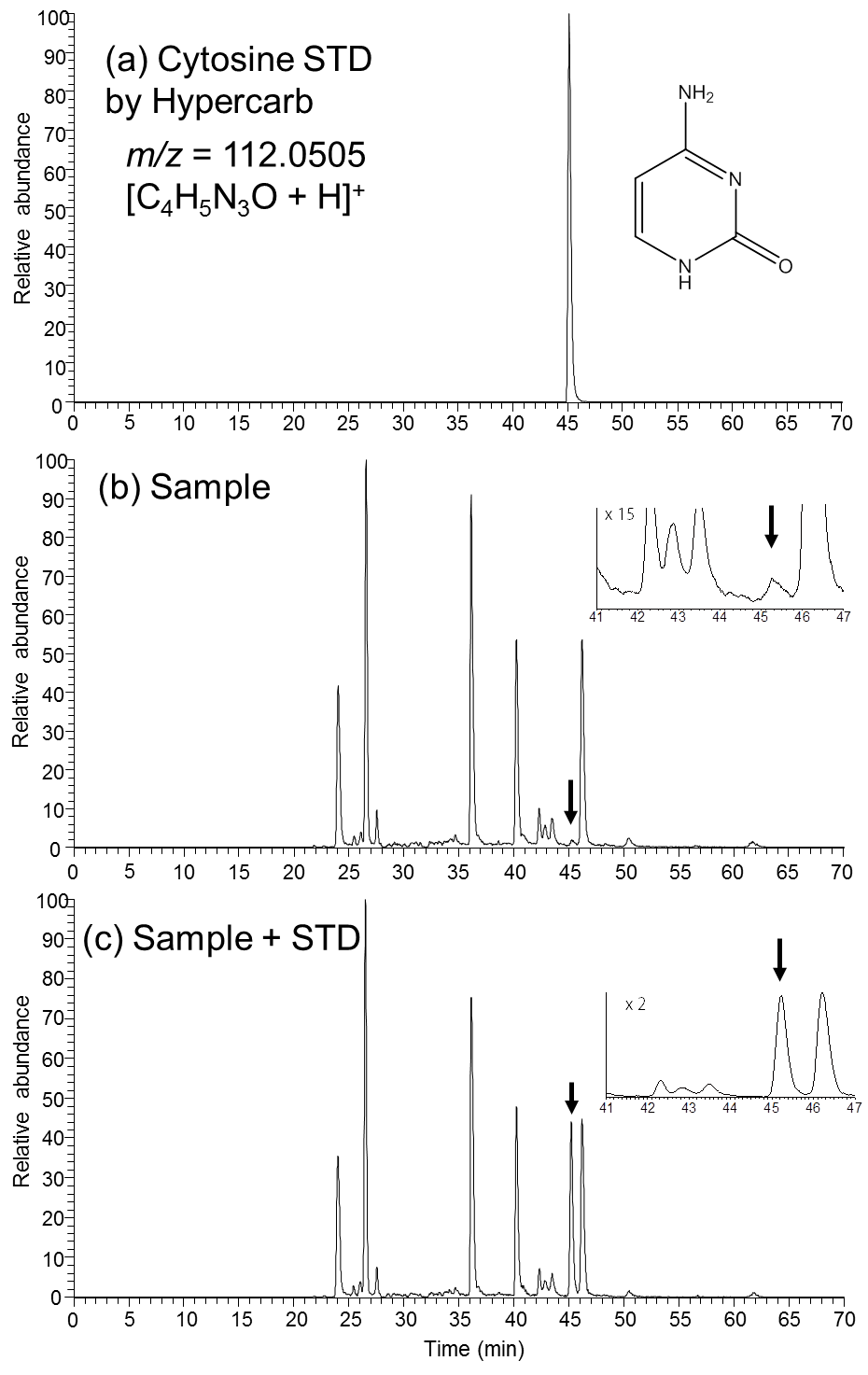


Supplementary Figure 3B. Identification of cytosine in the organic residues. Mass chromatograms of (a) the cytosine standard, (b) the analyte sample and (c) the co-injected mixture of cytosine standard and analyte sample at the *m/z* of 112.0505. A Hypercarb^TM^ separation column was used for the HPLC/HRMS analysis. The solid arrow indicates cytosine. The inset consists of a blow-up of the section of the spectrum between 41 and 47 min.


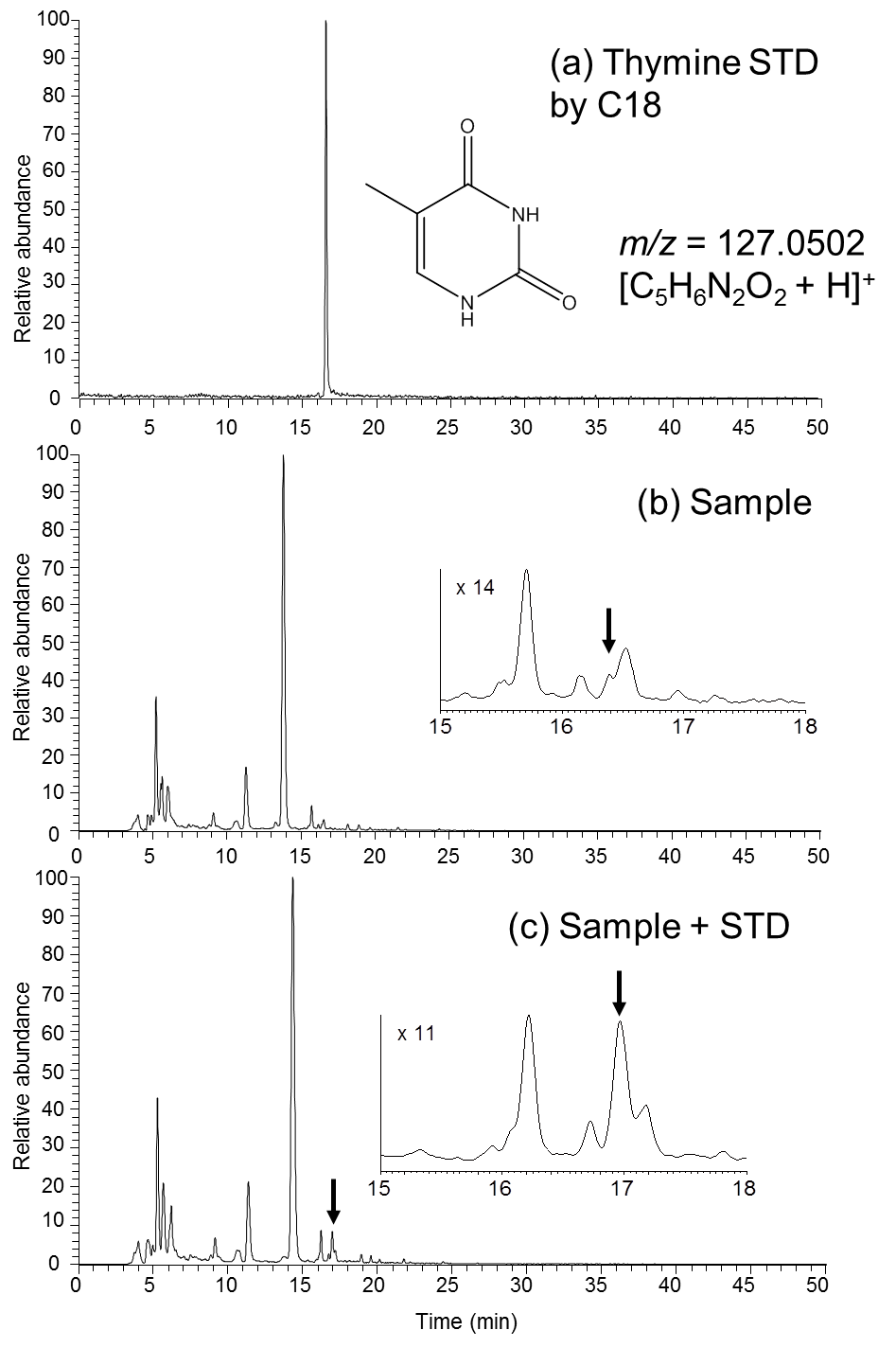


Supplementary Figure 4A. Identification of thymine in the organic residues. Mass chromatograms of (a) the thymine standard, (b) the analyte sample and (c) the co-injected mixture of thymine standard and analyte sample at the *m/z* of 127.0502. A C18 separation column was used for the HPLC/HRMS analysis. The solid arrow indicates thymine. The inset consists of a blow-up of the section of the spectrum between 15 and 18 min.


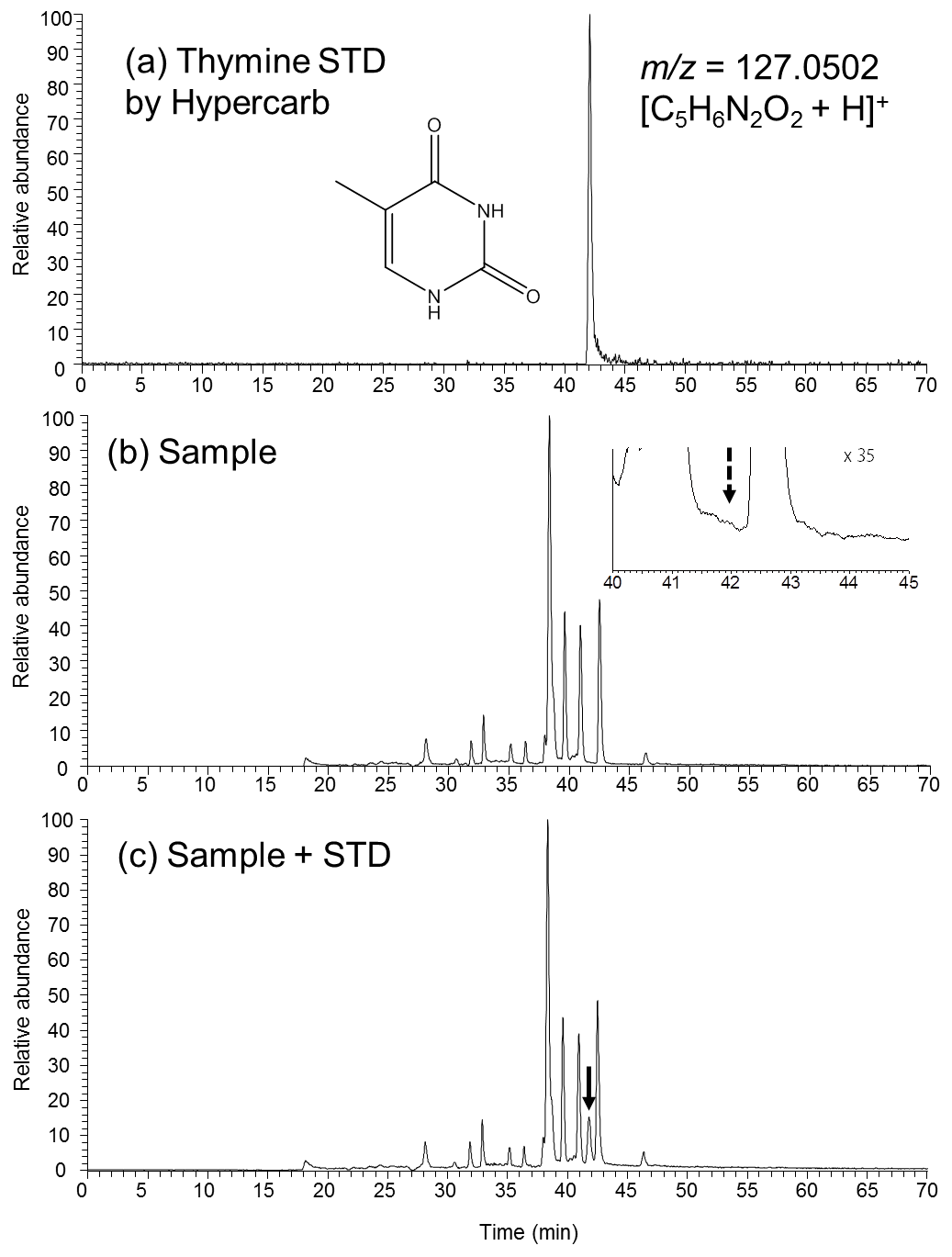


Supplementary Figure 4B. Identification of thymine in the organic residues. Mass chromatograms of (a) the thymine standard, (b) the analyte sample, and (c) the co-injected mixture of the thymine standard and the analyte sample at the *m/z* of 127.0502. A Hypercarb separation column was used on the analysis by HPLC. The dotted arrow in panel (b) indicates the possible presence of thymine in the analyte-only sample. The solid arrow indicates thymine detected in the co-injected sample. The inset shows an enlarged spectrum at 40 and 45 min.


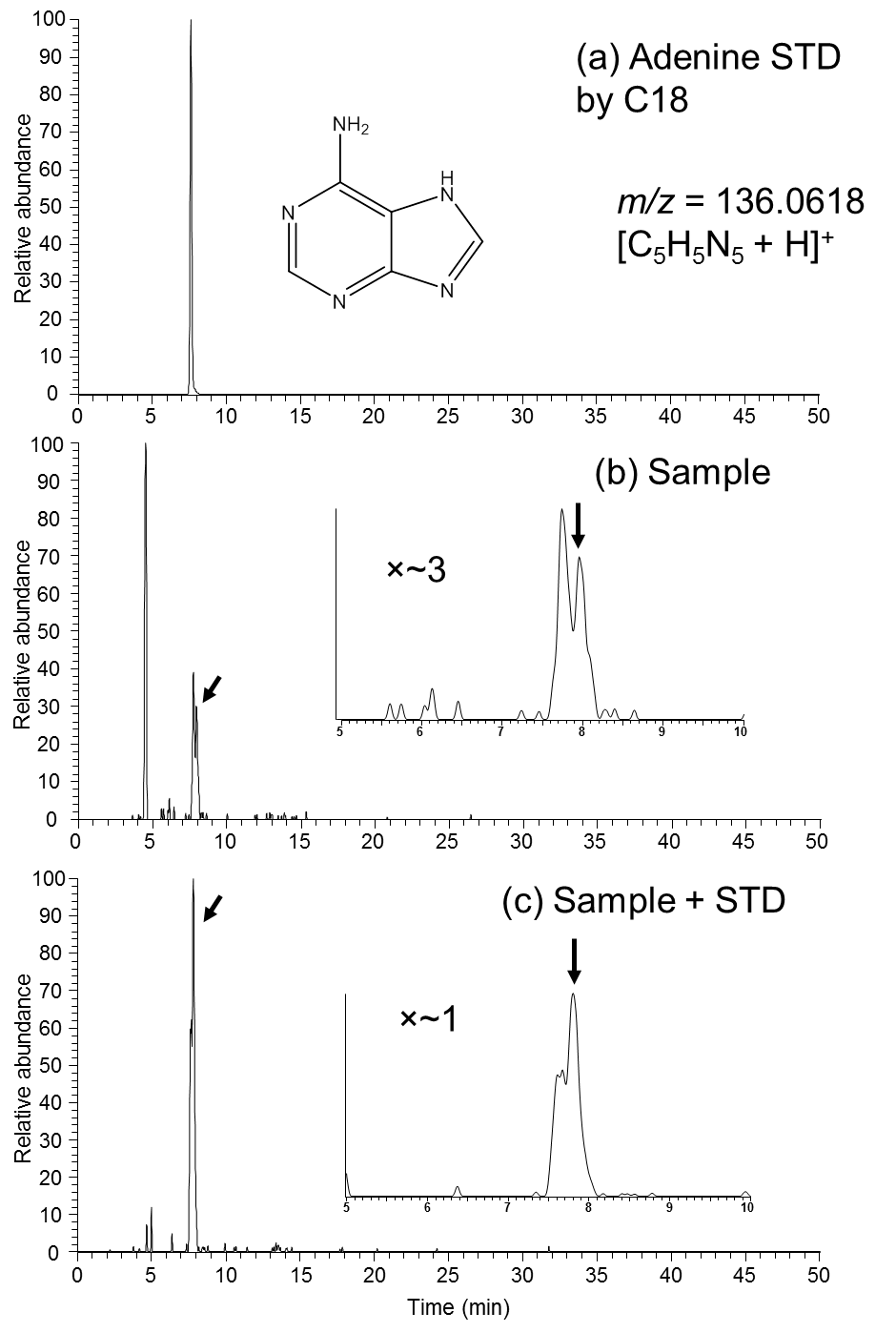


Supplementary Figure 5. Identification of adenine in the organic residues. Mass chromatograms of (a) the adenine standard, (b) the analyte sample and (c) the co-injected mixture of adenine standard and analyte sample at the *m/z* of 136.0618. A C18 separation column was used for the HPLC/HRMS analysis. The solid arrow indicates adenine. The inset consists of a blow-up of the section of the spectrum between 5 and 10 min.


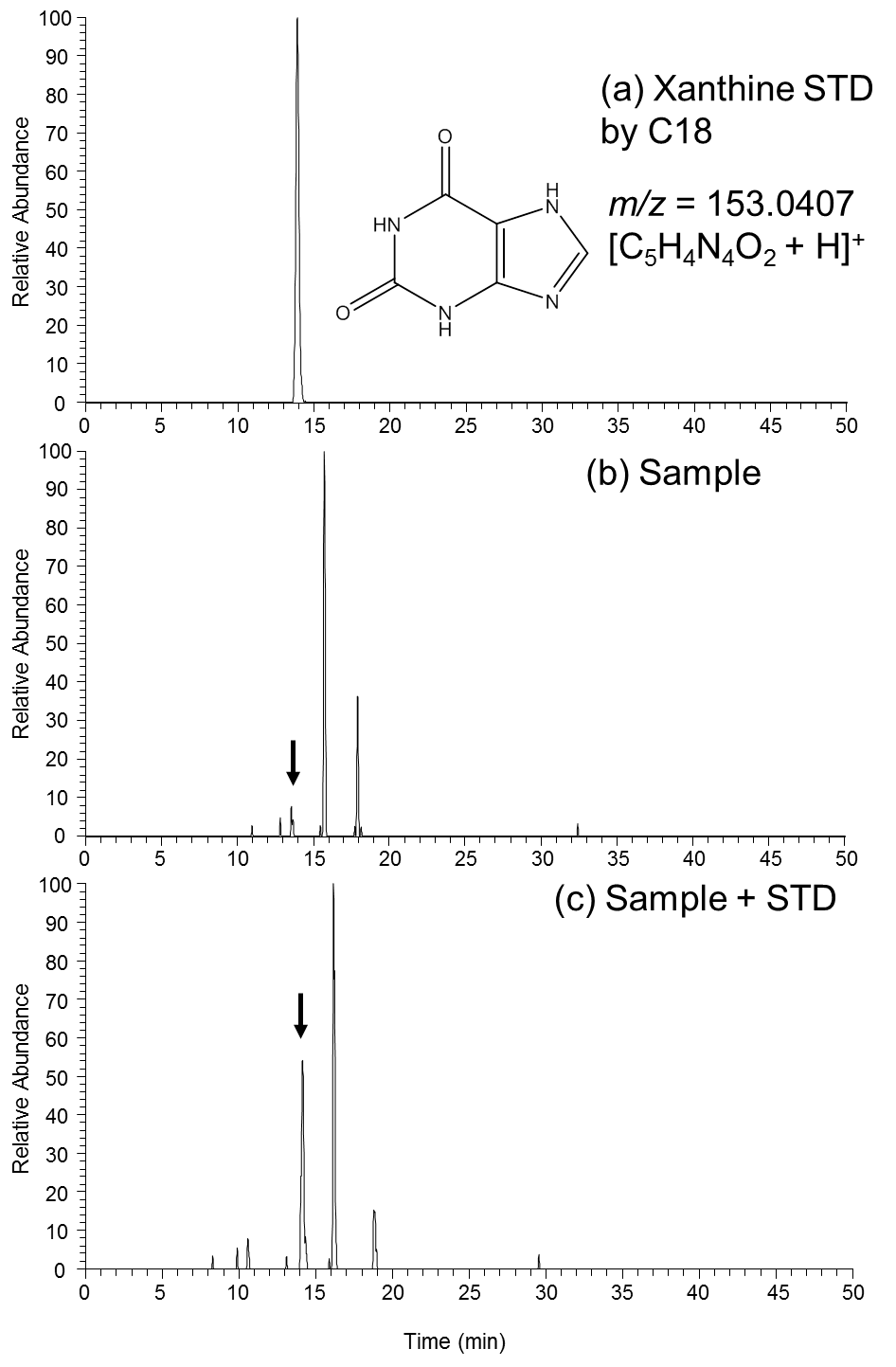


Supplementary Figure 6. Identification of xanthine in the organic residues. Mass chromatograms of (a) the xanthine standard, (b) the analyte sample and (c) the co-injected mixture of xanthine standard and analyte sample at the *m/z* of 153.0407. A C18 separation column was used for the HPLC/HRMS analysis. The solid arrow indicates xanthine.


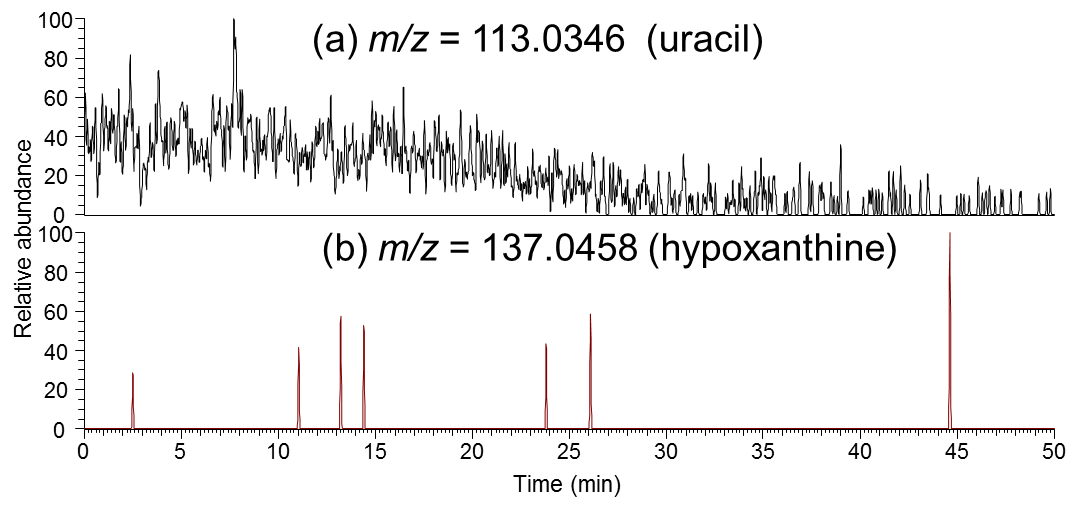


Supplementary Figure 7. Mass chromatograms of the procedural blank sample. A Hypercarb^TM^ separation column was used for the HPLC/HRMS analysis at a mass-to-charge ratio (*m/z*) of (a) 113.0346 and (b) 137.0458. These *m/z* values are those expected for protonated uracil and hypoxanthine, respectively. No peaks derived from the target molecules are present in this study


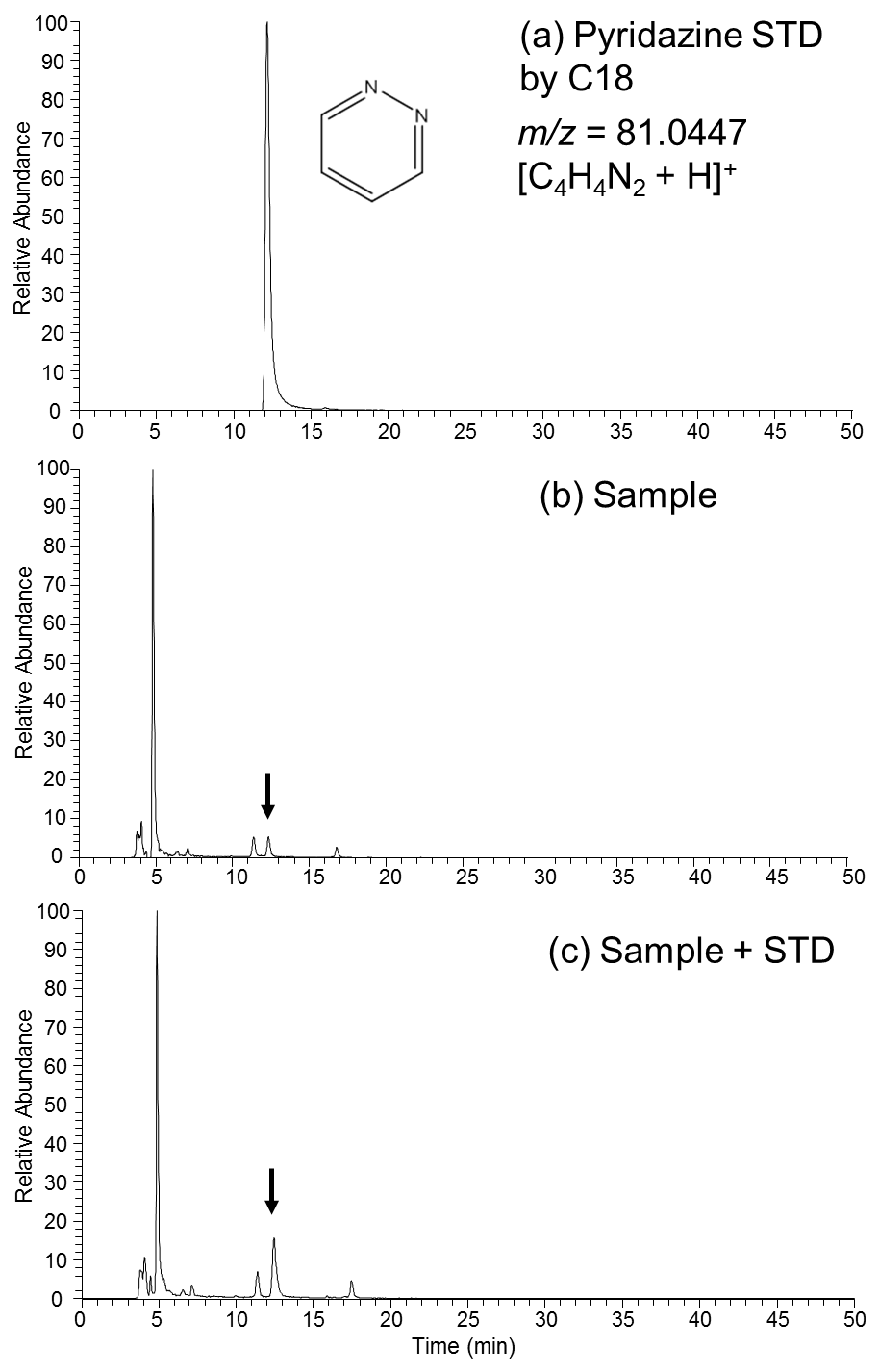


Supplementary Figure 8A. Identification of pyridazine in the organic residues. Mass chromatograms of (a) the pyridazine standard, (b) the analyte sample and (c) the co-injected mixture of pyridazine standard and analyte sample at the *m/z* of 81.0447. A C18 separation column was used for the HPLC/HRMS analysis. The solid arrow indicates pyridazine.


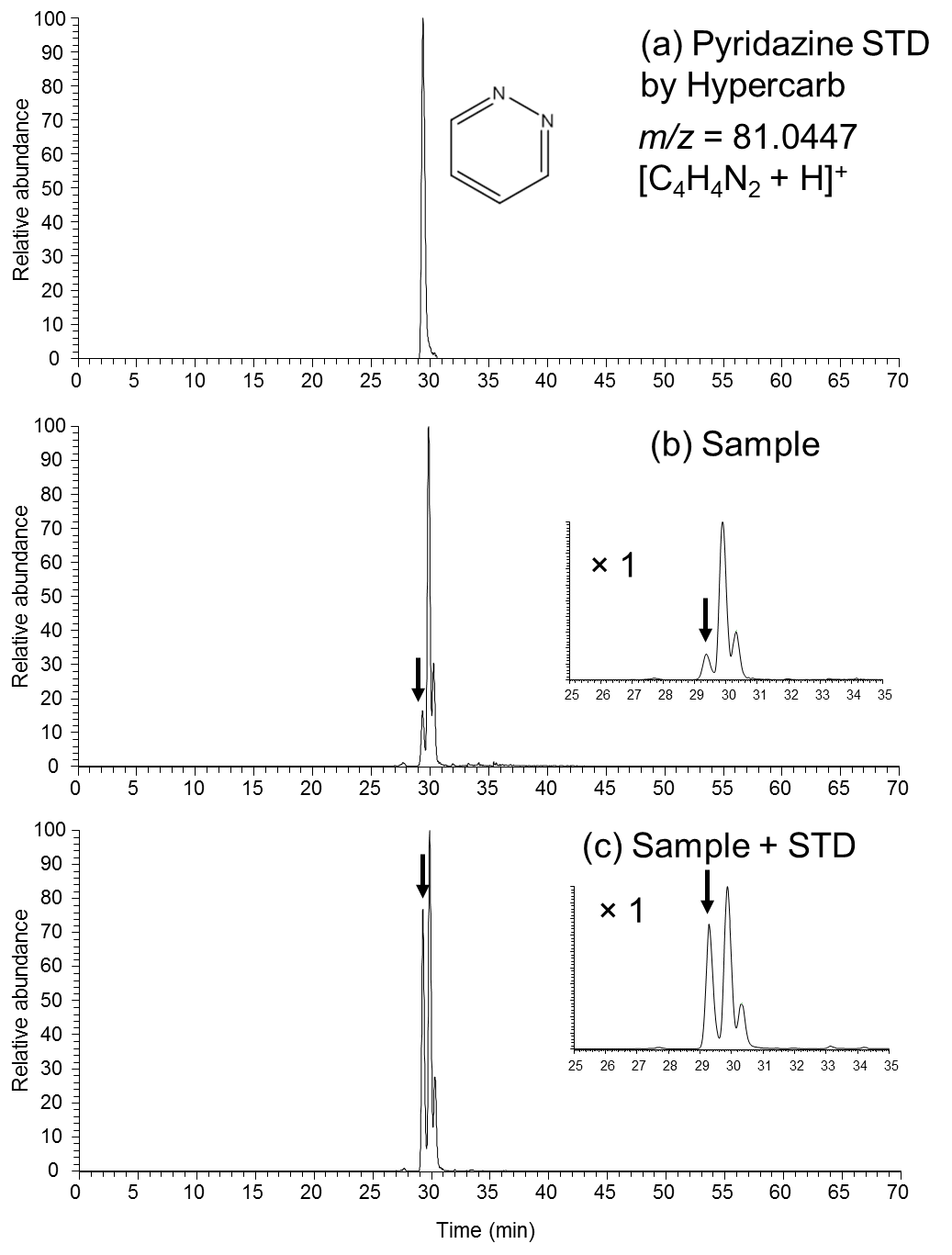


Supplementary Figure 8B. Identification of pyridazine in the organic residues. Mass chromatograms of (a) the pyridazine standard, (b) the analyte sample and (c) the co-injected mixture of pyridazine standard and analyte sample at the *m/z* of 81.0447. A Hypercarb^TM^ separation column was used for the HPLC/HRMS analysis. The solid arrow indicates pyridazine. The inset consists of a blow-up of the section of the spectrum between 25 and 35 min.


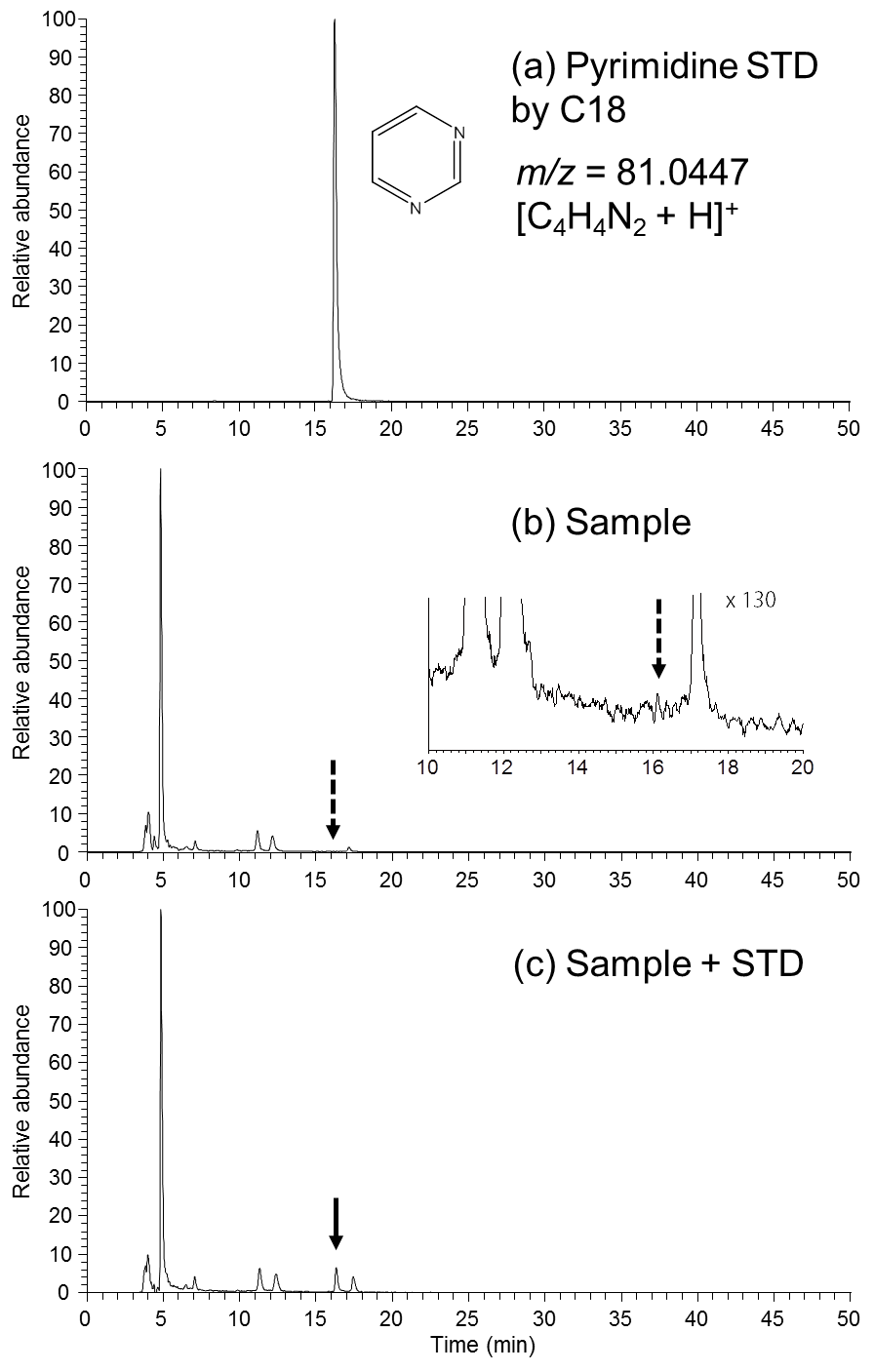


Supplementary Figure 9A. Identification of pyrimidine in the organic residues. Mass chromatograms of (a) the pyrimidine standard, (b) the analyte sample, and (c) the co-injected mixture of the pyrimidine standard and the analyte sample at the *m/z* of 81.0447. A C18 separation column was used on the analysis by HPLC. The dotted arrow in panel (b) indicates the possible presence of pyrimidine in the analyte-only sample. The solid arrow indicates the presence of pyrimidine in the co-injected sample. The inset shows an enlarged spectrum at 10 and 20 min.


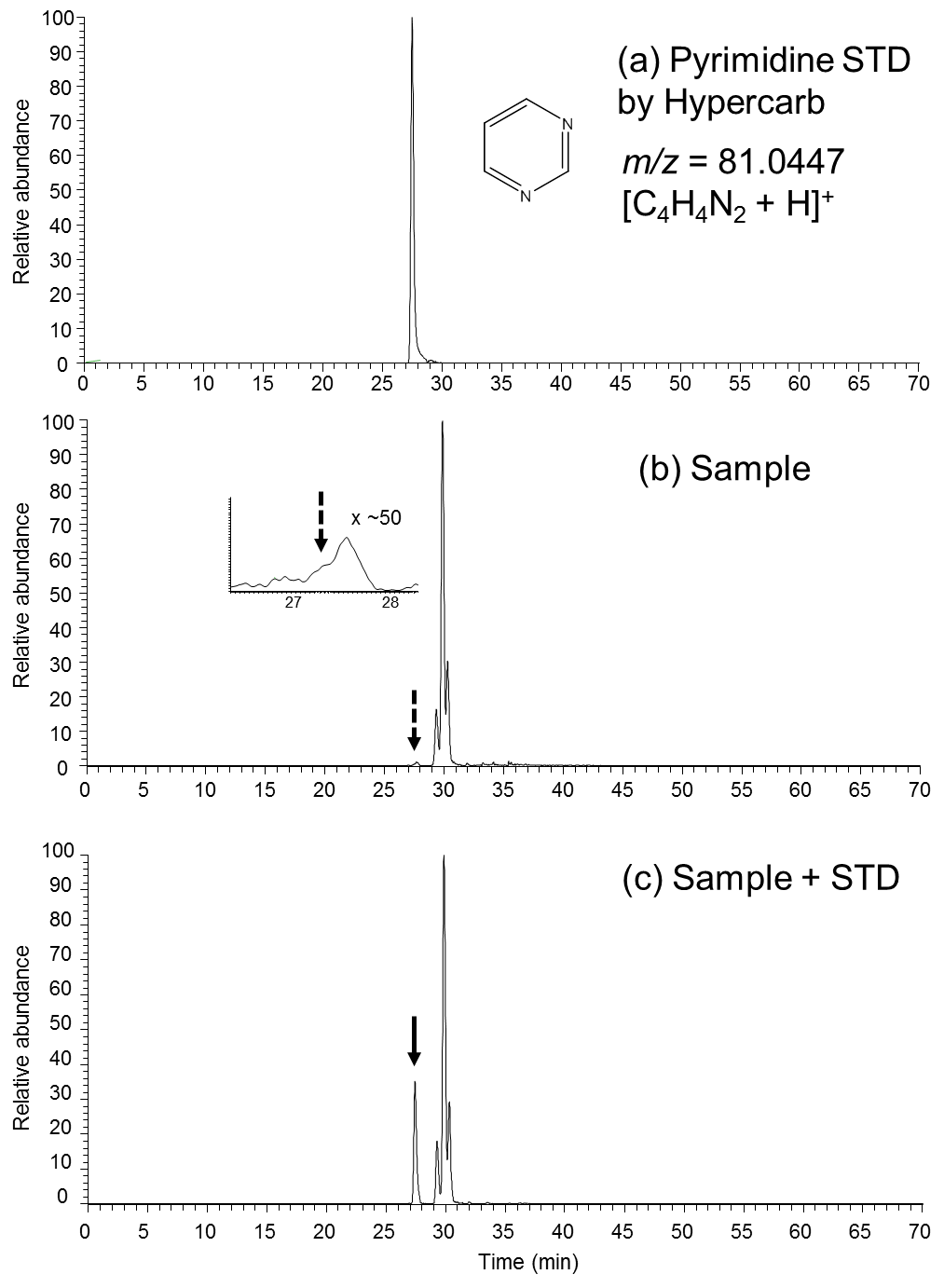


Supplementary Figure 9B. Identification of pyrimidine in the organic residues. Mass chromatograms of (a) the pyrimidine standard, (b) the analyte sample, and (c) the co-injected mixture of the pyrimidine standard and the analyte sample at the *m/z* of 81.0447. A Hypercarb separation column was used on the analysis by HPLC. The dotted arrow in panel (b) indicates the possible presence of pyrimidine in the analyte-only sample. The solid arrow indicates the presence of pyrimidine in the co-injected sample. The inset shows an enlarged spectrum at 27.5 to 28.2 min.


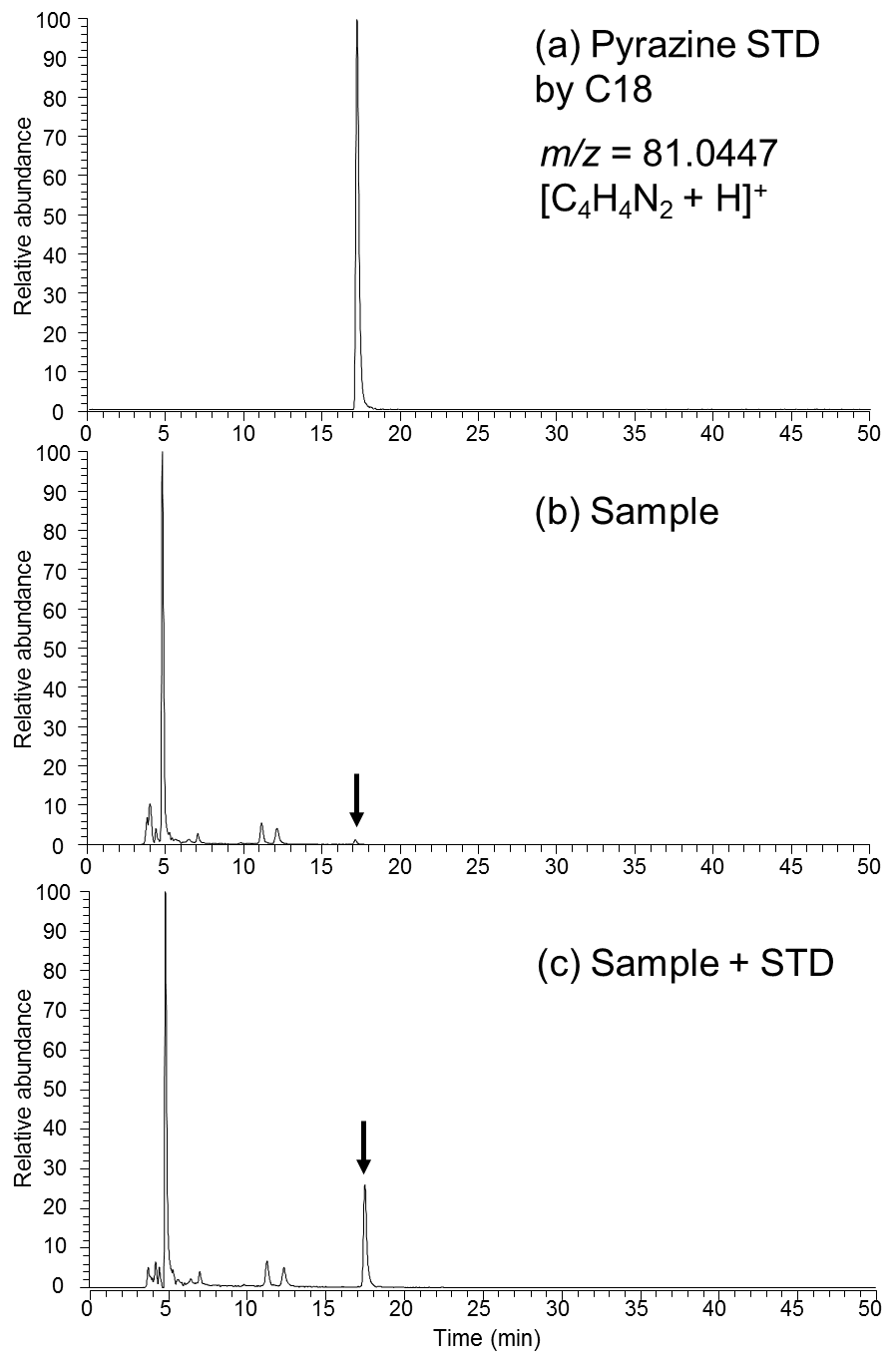


Supplementary Figure 10A. Identification of pyrazine in the organic residues. Mass chromatograms of (a) the pyrazine standard, (b) the analyte sample and (c) the co-injected mixture of pyrazine standard and analyte sample at the *m/z* of 81.0447. A C18 separation column was used for the HPLC/HRMS analysis. The solid arrow indicates pyrazine.


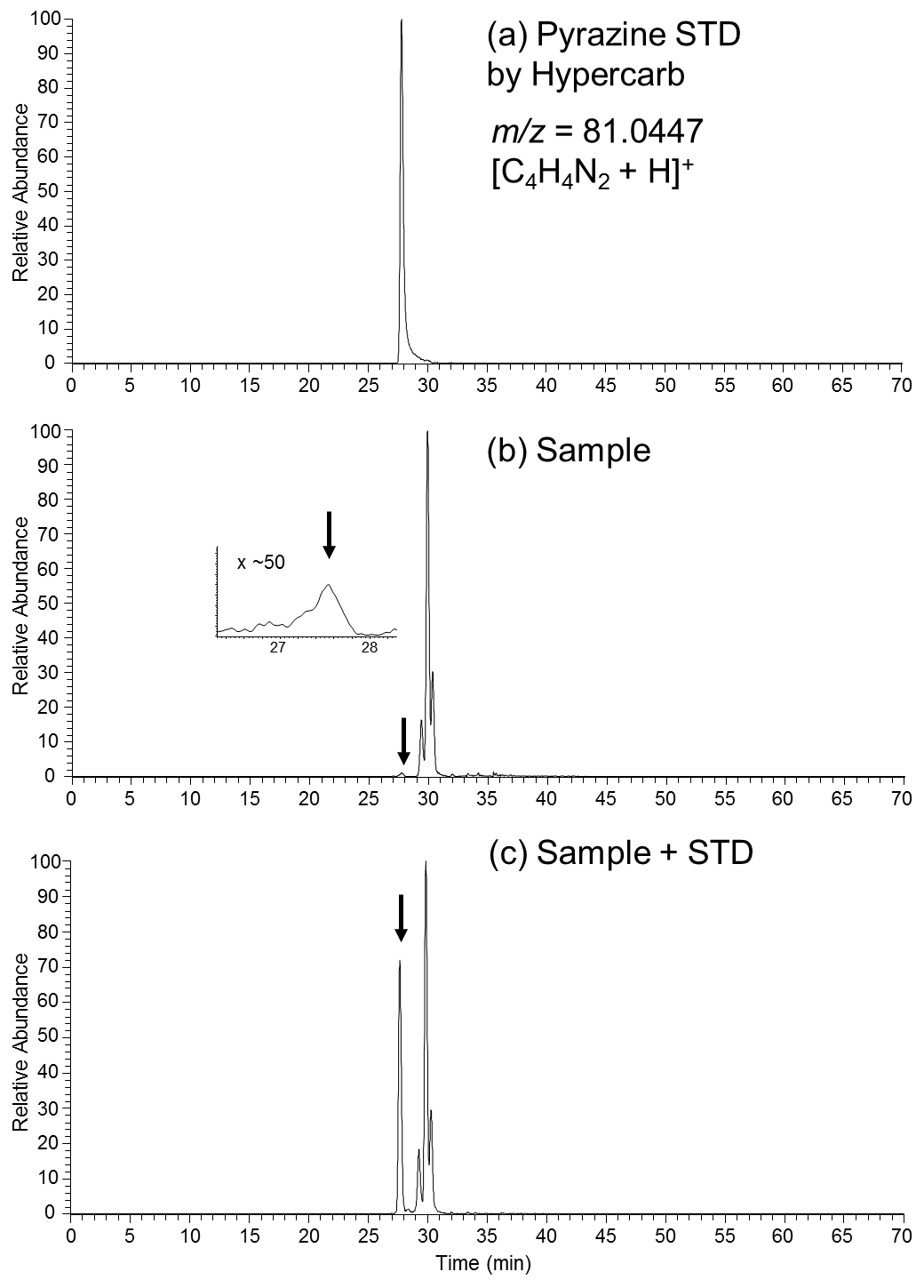


Supplementary Figure 10B. Identification of pyrazine in the organic residues. Mass chromatograms of (a) the pyrazine standard, (b) the analyte sample and (c) the co-injected mixture of pyrazine standard and analyte sample at the *m/z* of 81.0447. A Hypercarb^TM^ separation column was used for the HPLC/HRMS analysis. The solid arrow indicates pyrazine. The inset consists of a blow-up of the section of the spectrum between 27.5 and 28.2 min.


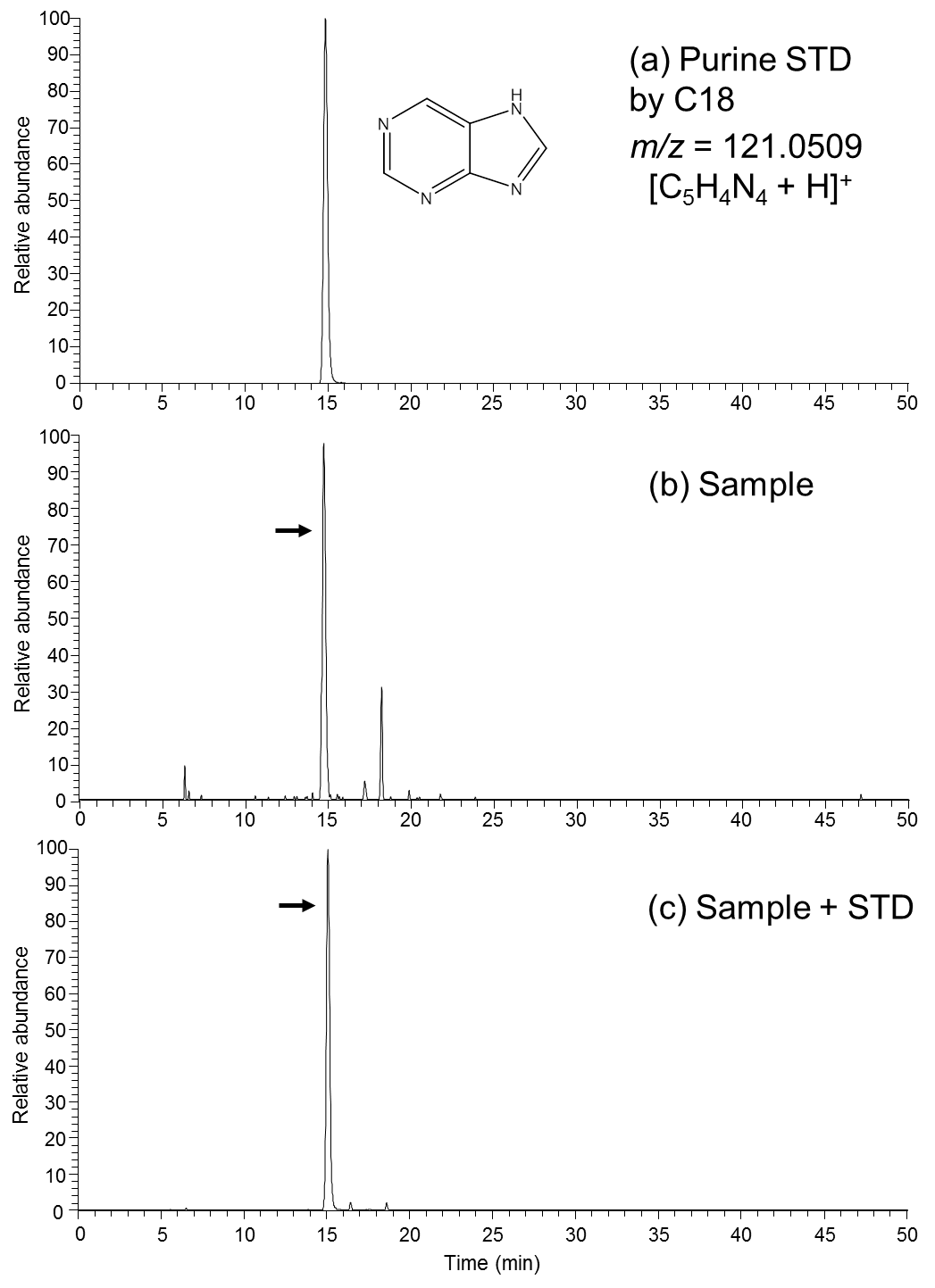


Supplementary Figure 11A. Identification of purine in the organic residues. Mass chromatograms of (a) the purine standard, (b) the analyte sample and (c) the co-injected mixture of purine standard and analyte sample at the *m/z* of 121.0509. A C18 separation column was used for the HPLC/HRMS analysis. The solid arrow indicates purine.


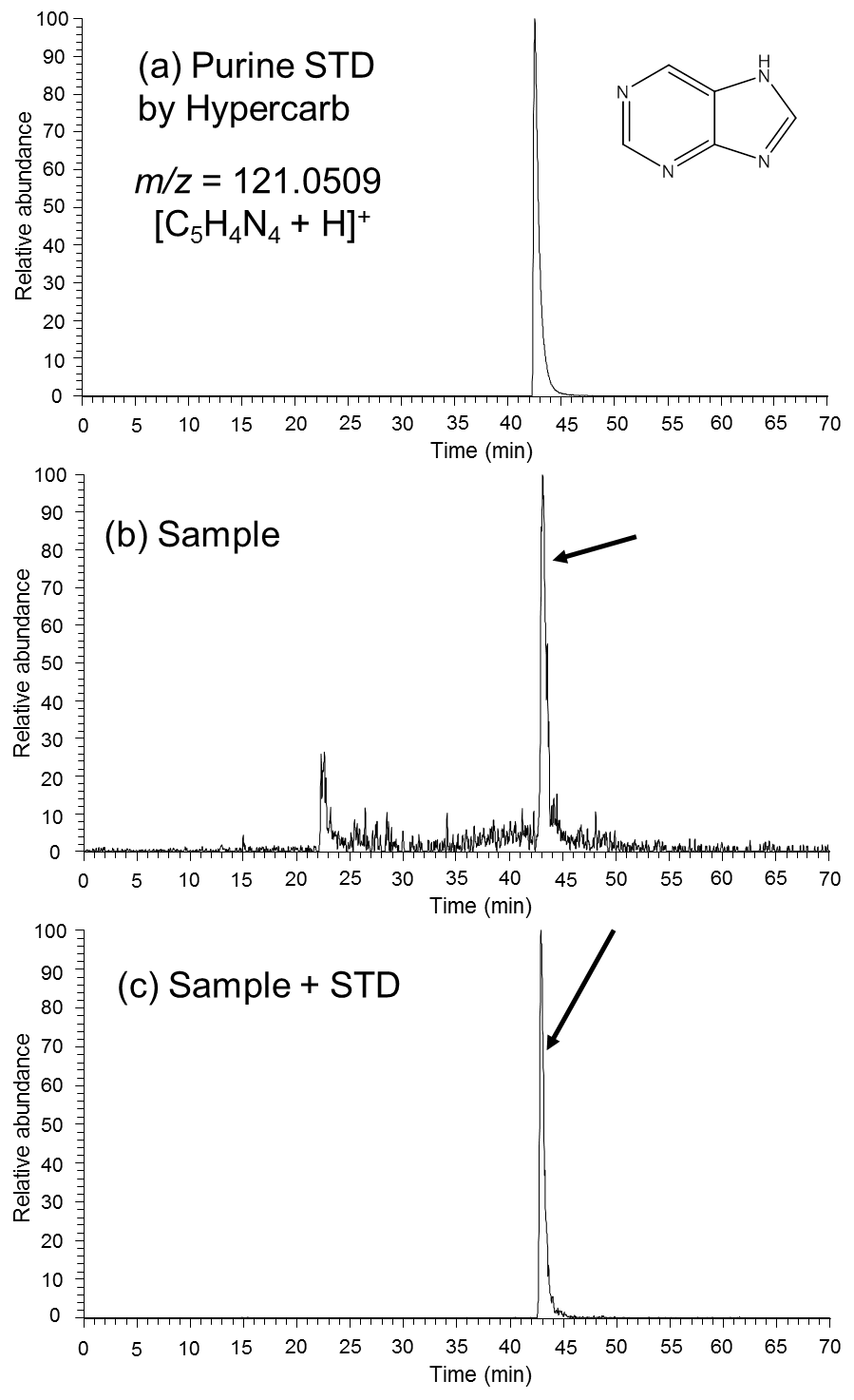


Supplementary Figure 11B. Identification of purine in the organic residues. Mass chromatograms of (a) the purine standard, (b) the analyte sample and (c) the co-injected mixture of purine standard and analyte sample at the *m/z* of 121.0509. A Hypercarb^TM^ separation column was used for the HPLC/HRMS analysis. The solid arrow indicates purine.


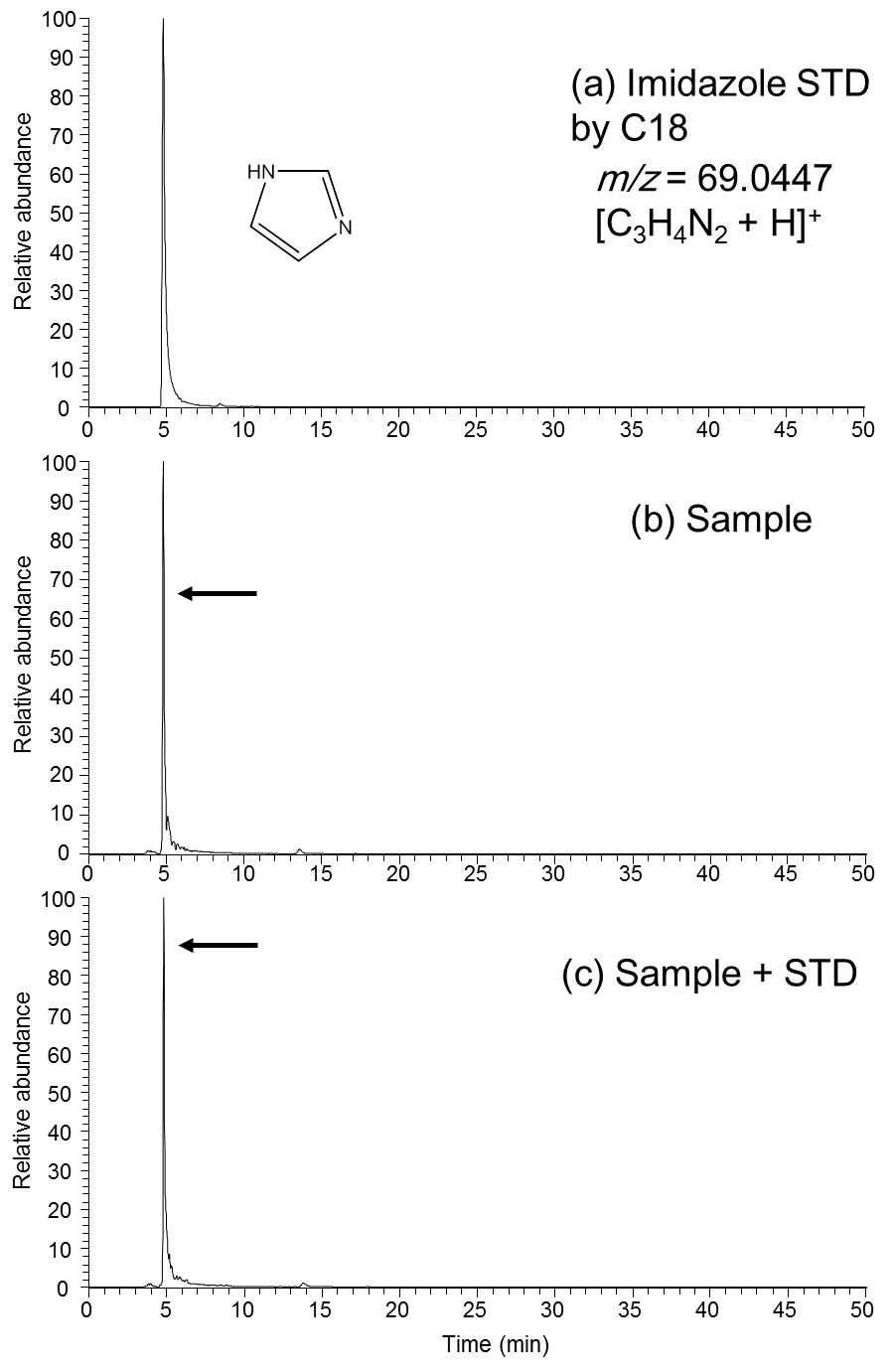


Supplementary Figure 12A. Identification of imidazole in the organic residues. Mass chromatograms of (a) the imidazole standard, (b) the analyte sample and (c) the co-injected mixture of imidazole standard and analyte sample at the *m/z* of 69.0447. A C18 separation column was used for the HPLC/HRMS analysis. The solid arrow indicates imidazole.


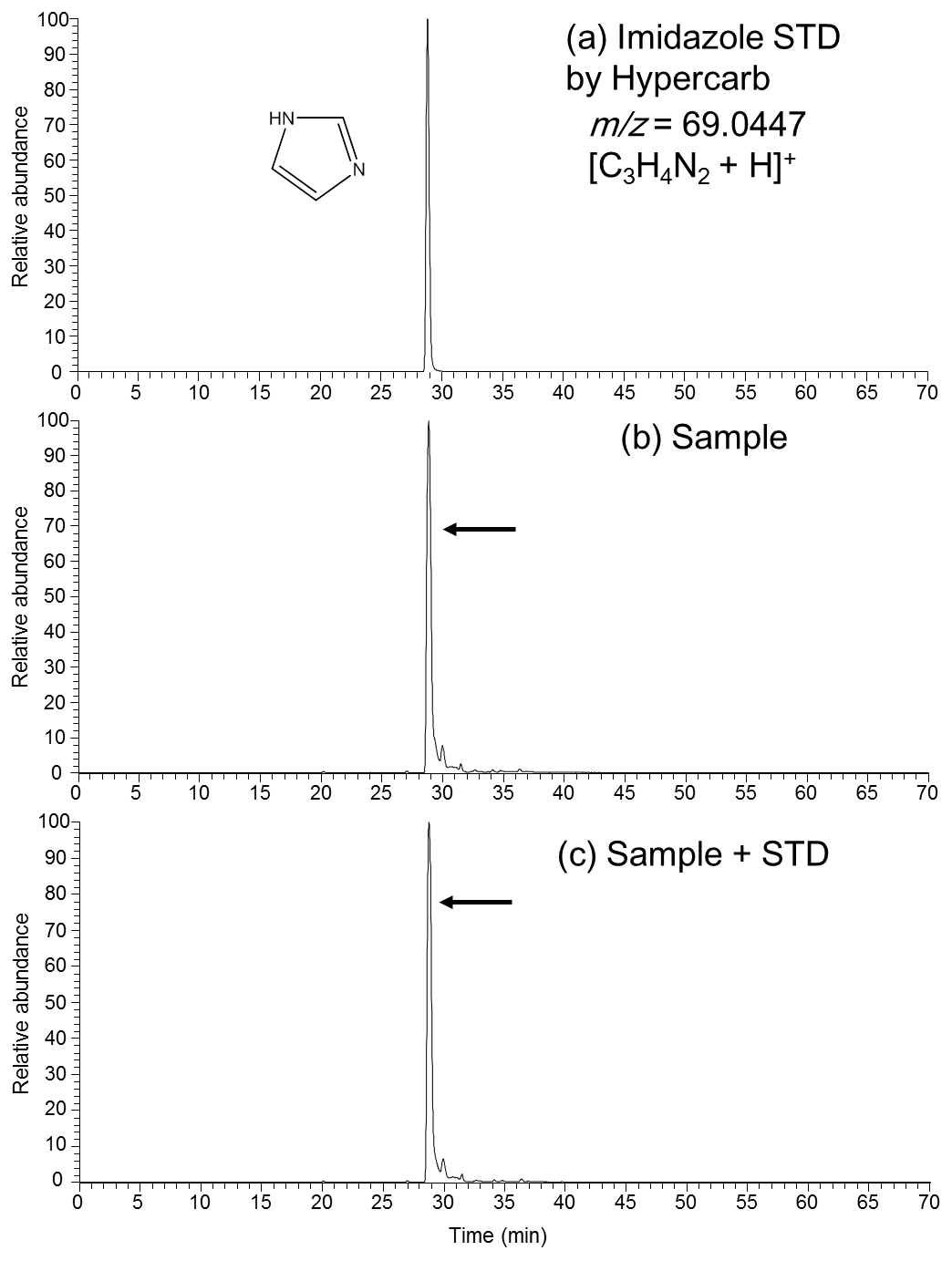


Supplementary Figure 12B. Identification of imidazole in the organic residues. Mass chromatograms of (a) the imidazole standard, (b) the analyte sample and (c) the co-injected mixture of imidazole standard and analyte sample at the *m/z* of 69.0447. A Hypercarb^TM^ separation column was used for the HPLC/HRMS analysis. The solid arrow indicates imidazole.


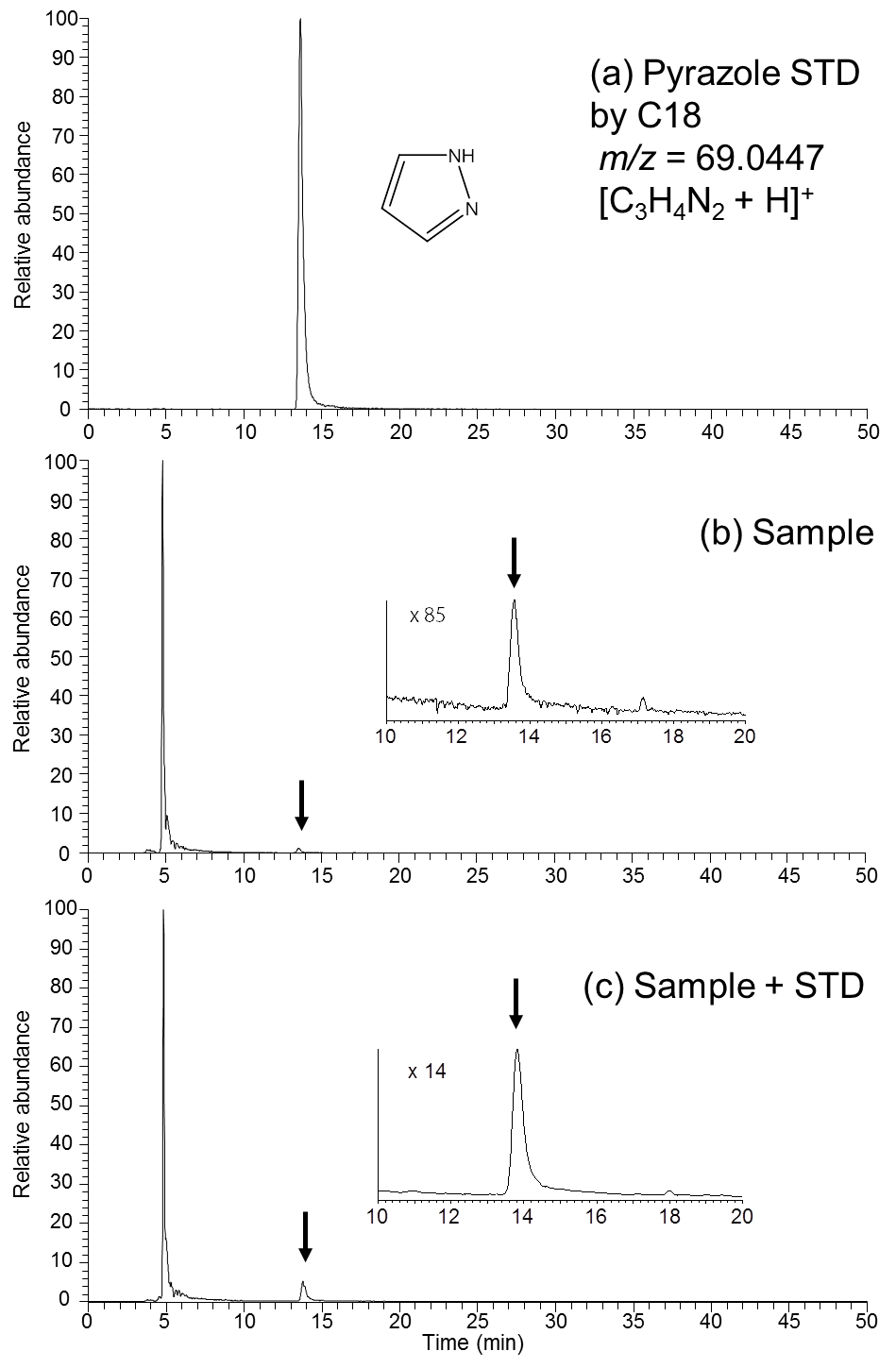


Supplementary Figure 13A. Identification of pyrazole in the organic residues. Mass chromatograms of (a) the pyrazole standard, (b) the analyte sample and (c) the co-injected mixture of pyrazole standard and analyte sample at the *m/z* of 69.0447. A C18 separation column was used for the HPLC/HRMS analysis. The solid arrow indicates pyrazole. The inset shows an enlarged spectrum at 10 to 20 min.


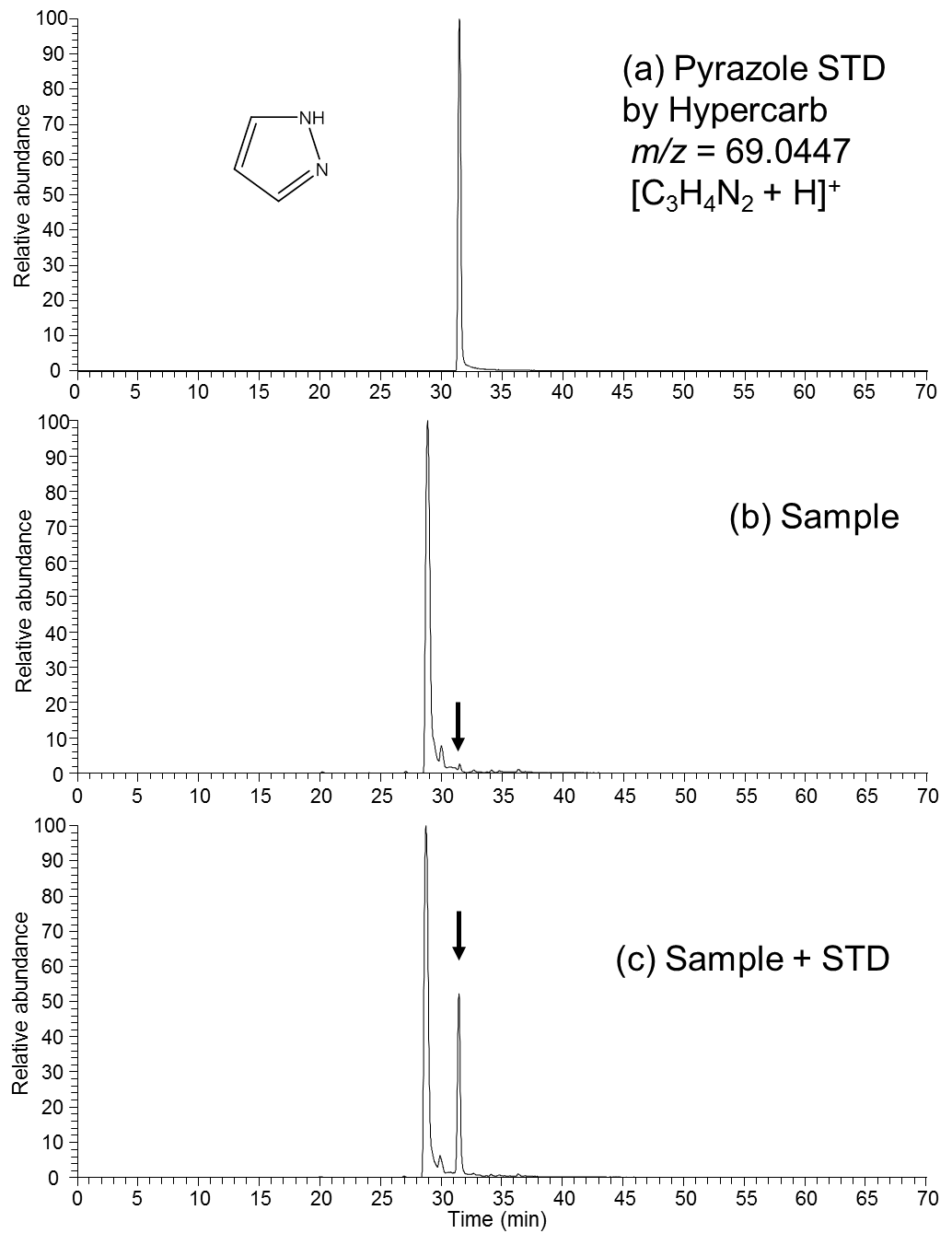


Supplementary Figure 13B. Identification of pyrazole in the organic residues. Mass chromatograms of (a) the pyrazole standard, (b) the analyte sample and (c) the co-injected mixture of pyrazole standard and analyte sample at the *m/z* of 69.0447. A Hypercarb^TM^ separation column was used for the HPLC/HRMS analysis. The solid arrow indicates pyrazole.


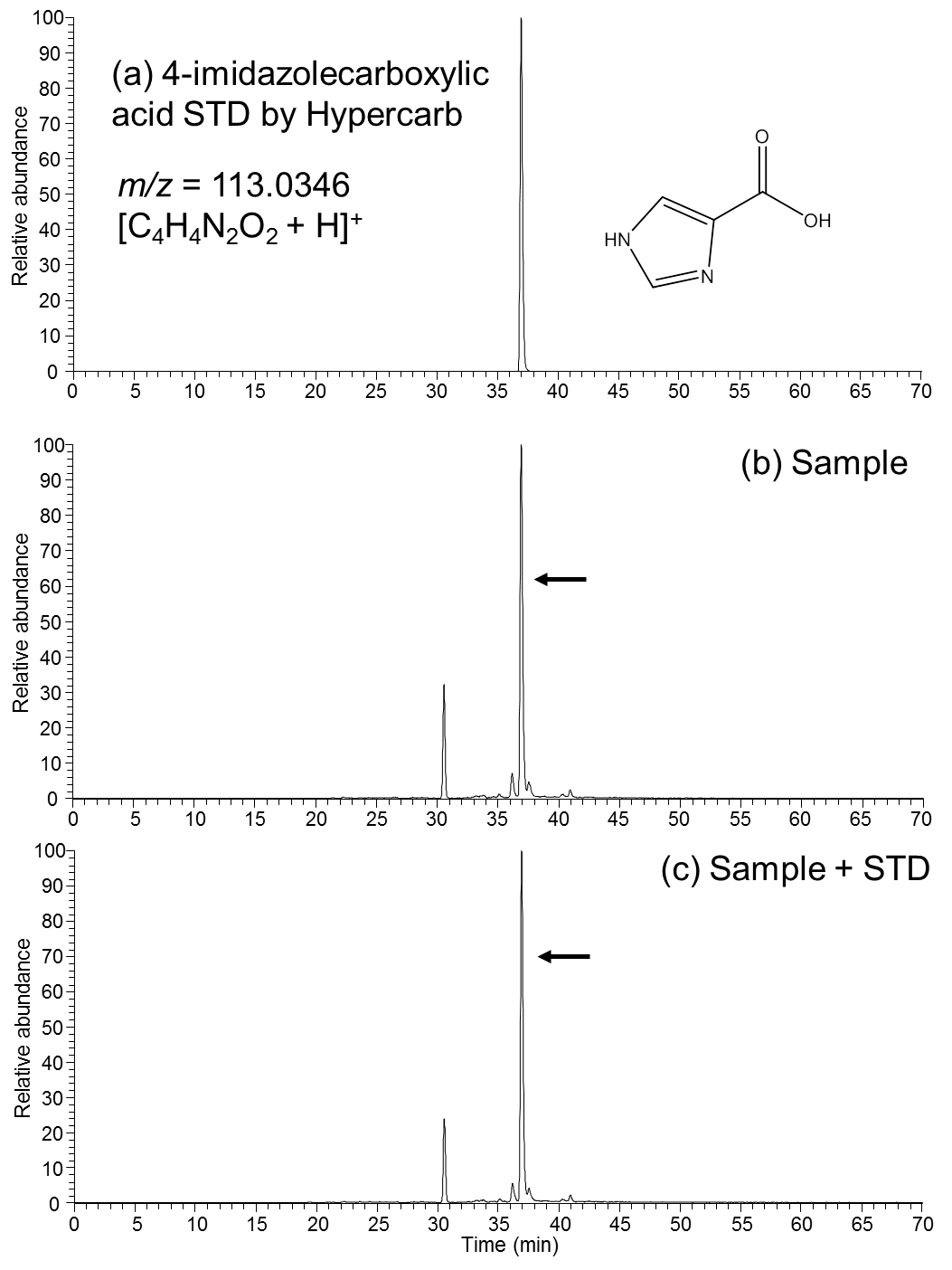


Supplementary Figure 14. Identification of 4-imidazolecarboxylic acid in the organic residues. Mass chromatograms of (a) the 4-imidazolecarboxylic acid standard, (b) the analyte sample and (c) the co-injected mixture of 4-imidazolecarboxylic acid standard and analyte sample at the *m/z* of 113.0346. A Hypercarb^TM^ separation column was used for the HPLC/HRMS analysis. The solid arrow indicates 4-imidazolecarboxylic acid.


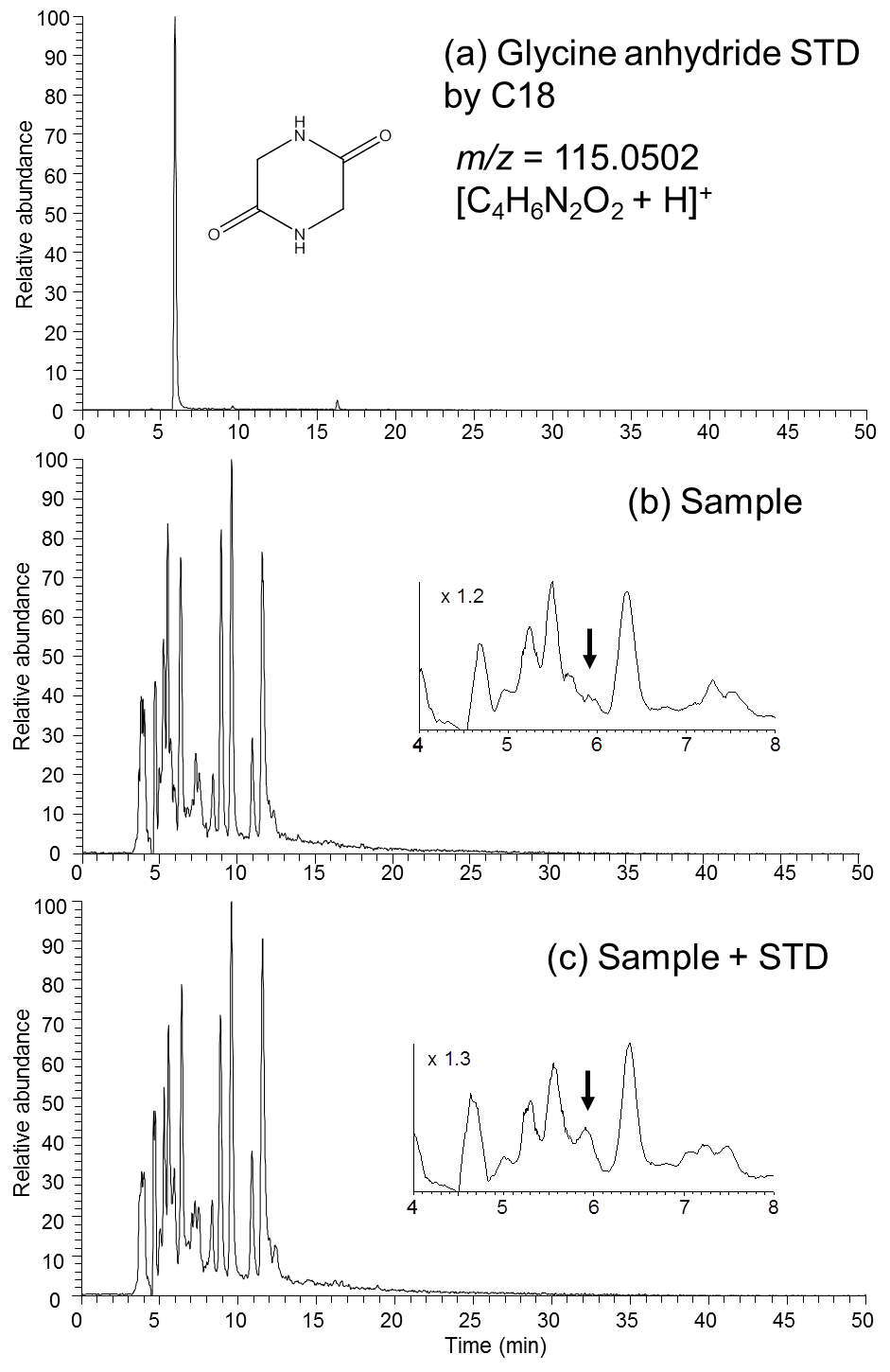


Supplementary Figure 15A. Identification of glycine anhydride in the organic residues. Mass chromatograms of (a) the glycine anhydride standard, (b) the analyte sample and (c) the co-injected mixture of glycine anhydride standard and analyte sample at the *m/z* of 115.0502. A C18 separation column was used for the HPLC/HRMS analysis. The solid arrow indicates glycine anhydride. The inset consists of a blow-up of the section of the spectrum between 4 and 8 min.


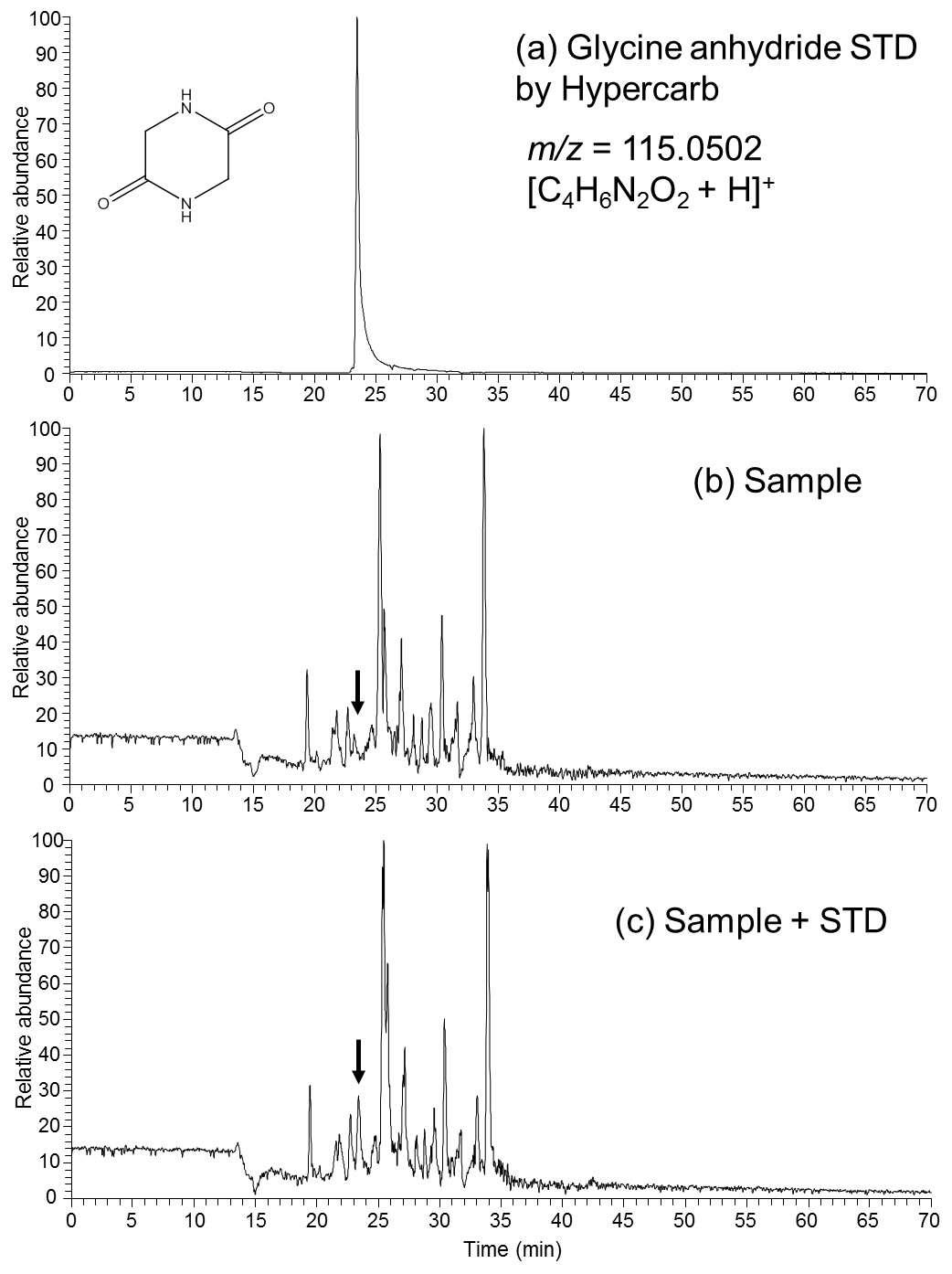


Supplementary Figure 15B. Identification of glycine anhydride in the organic residues. Mass chromatograms of (a) the glycine anhydride standard, (b) the analyte sample and (c) the co-injected mixture of glycine anhydride standard and analyte sample at the *m/z* of 115.0502. A Hypercarb^TM^ separation column was used for the HPLC/HRMS analysis. The solid arrow indicates glycine anhydride.


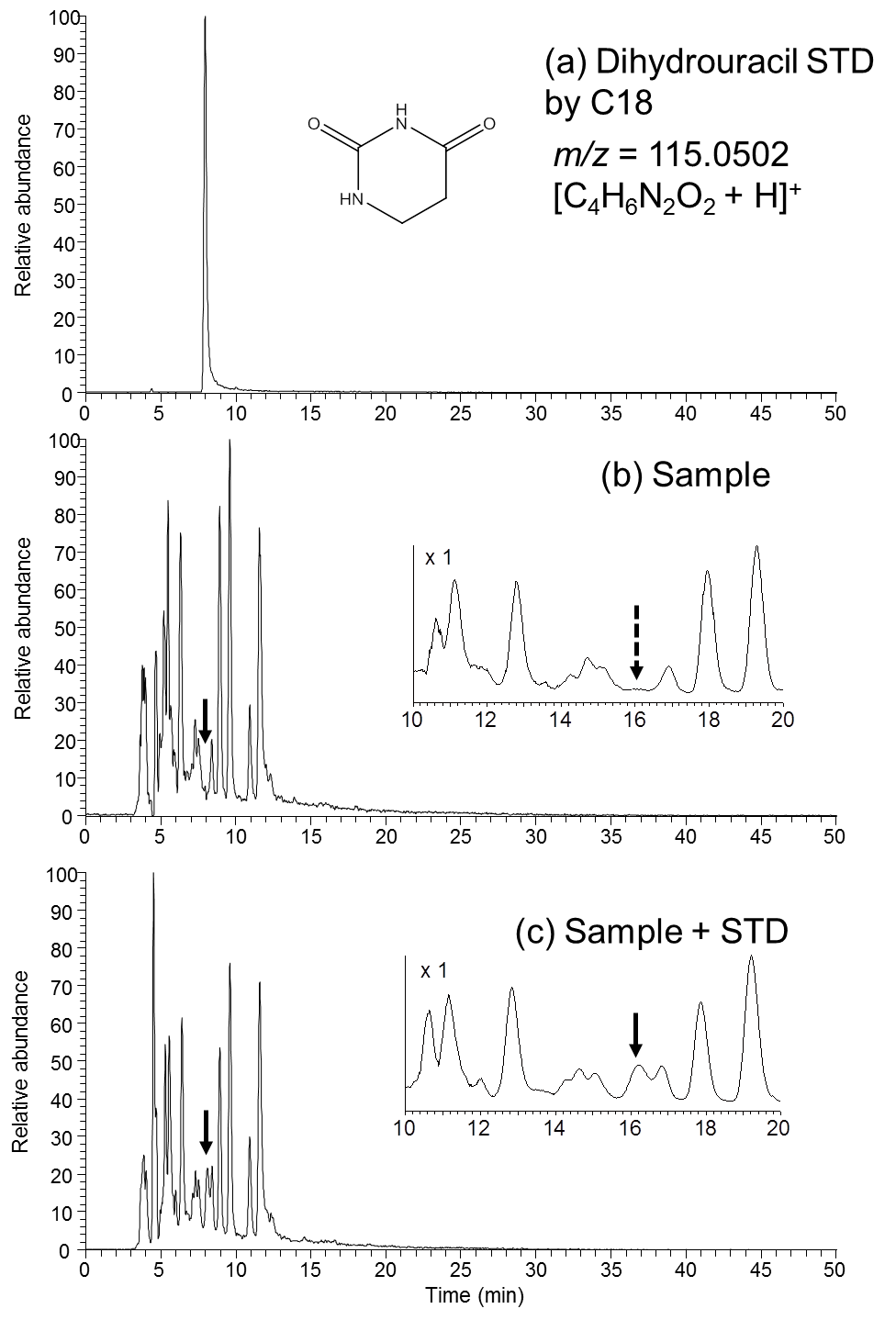


Supplementary Figure 16A. Identification of dihydrouracil in the organic residues. Mass chromatograms of (a) the dihydrouracil standard, (b) the analyte sample, and (c) the co-injected mixture of the dihydrouracil standard and the analyte sample at the *m/z* of 115.0502. A C18 separation column was used on the analysis by HPLC. The dotted arrow in panel (b) indicates the possible presence of dihydrouracil in the analyte-only sample. The solid arrow indicates dihydrouracil present in the co-injected samples. The inset shows an enlarged spectrum at 10 to 20 min.


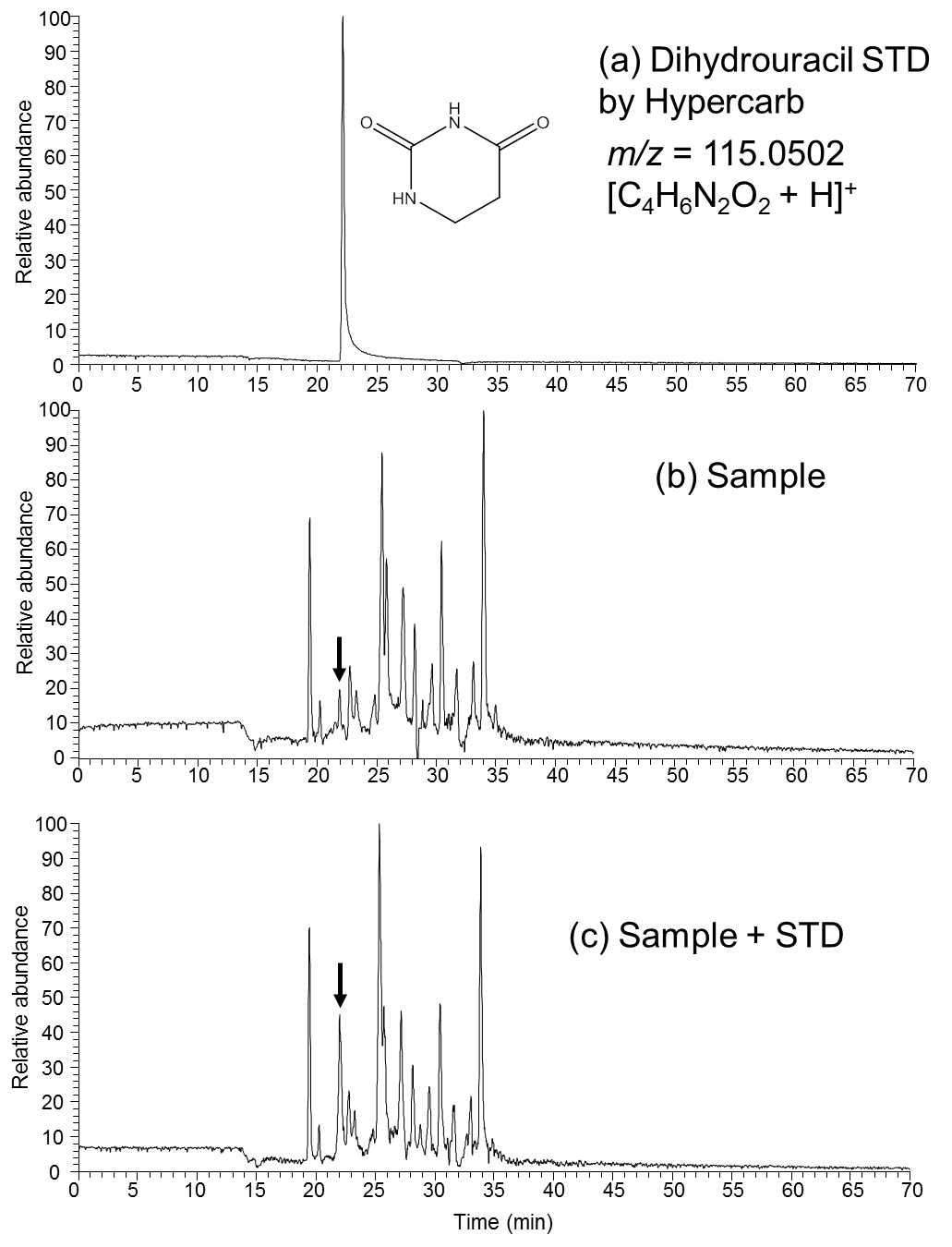


Supplementary Figure 16B. Identification of dihydrouracil in the organic residues. Mass chromatograms of (a) the dihydrouracil standard, (b) the analyte sample and (c) the co-injected mixture of dihydrouracil standard and analyte sample at the *m/z* of 115.0502. A Hypercarb^TM^ separation column was used for the HPLC/HRMS analysis. The solid arrow indicates dihydrouracil.


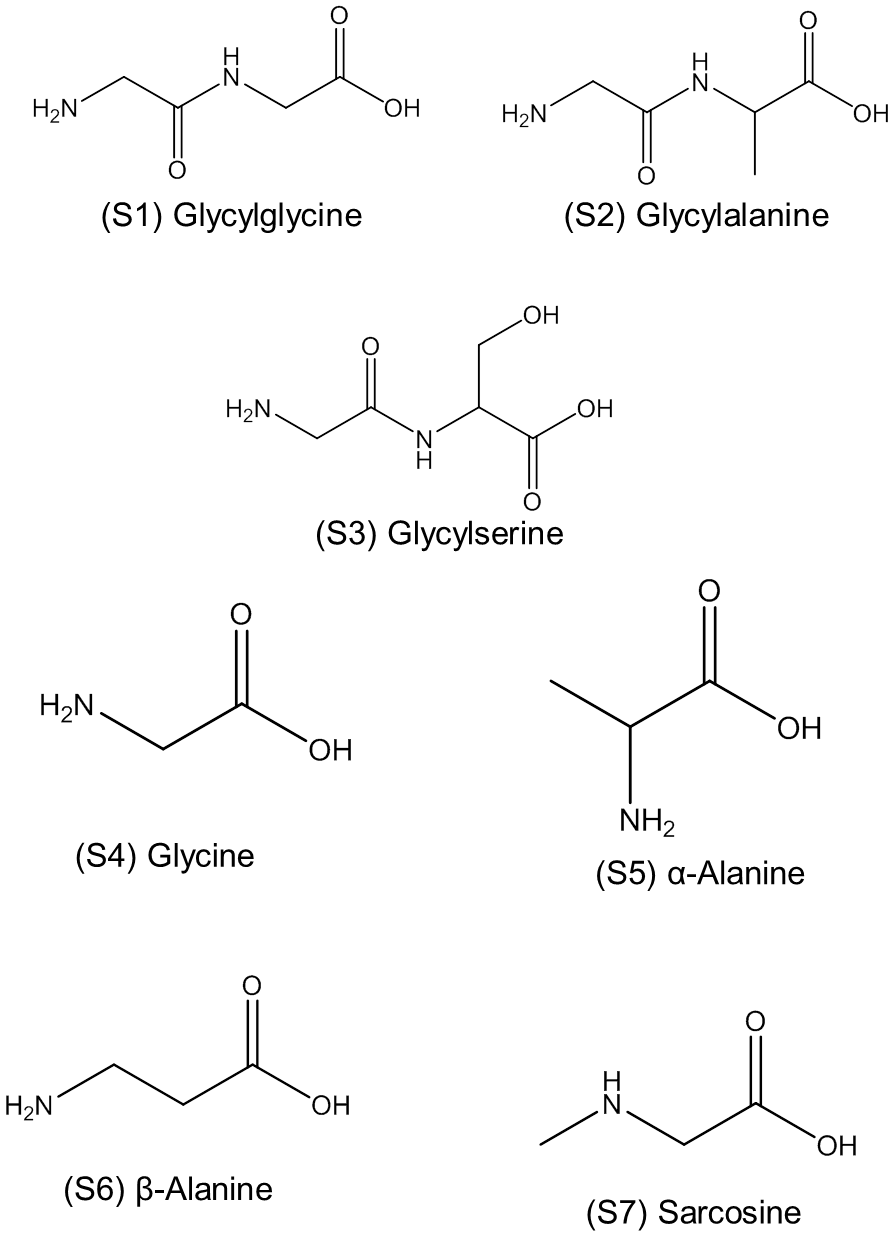


Supplementary Figure 17. Names and structures of the dipeptides and amino acids detected in the organic residues. The numbers in parentheses are used to identify the relevant compounds in Supplementary Table 1, where the yield of each species is reported.


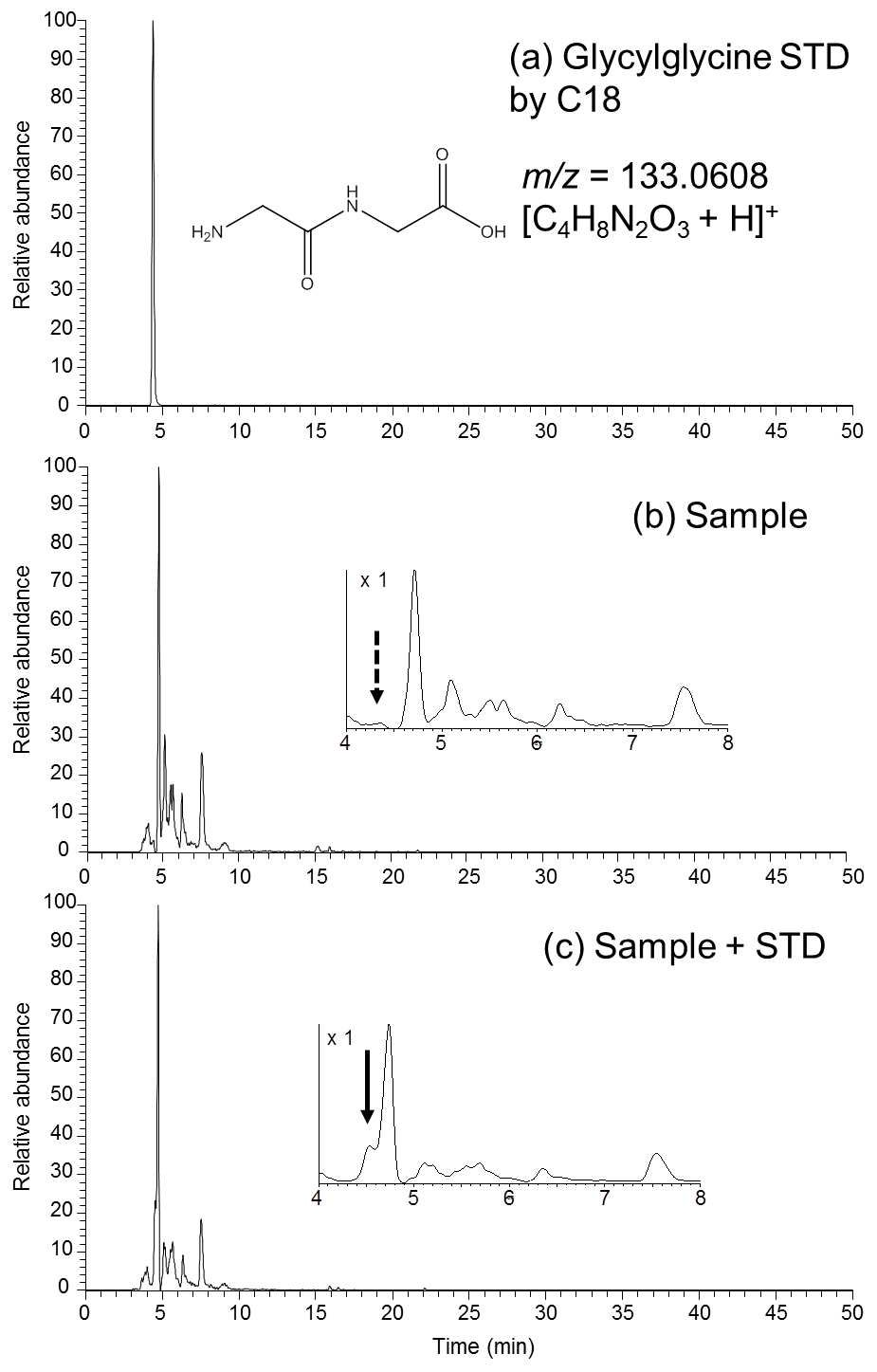


Supplementary Figure 18A. Identification of glycylglycine in the organic residues. Mass chromatograms of (a) the glycylglycine standard, (b) the analyte sample, and (c) the co-injected mixture of the glycylglycine standard and the analyte sample at the *m/z* of 133.0608. A C18 separation column was used on the analysis by HPLC. The dotted arrow indicates the possible presence of glycylglycine in the analyte-only sample. The solid arrow indicates glycylglycine present in the samples. The inset shows an enlarged spectrum at 4 to 8 min.


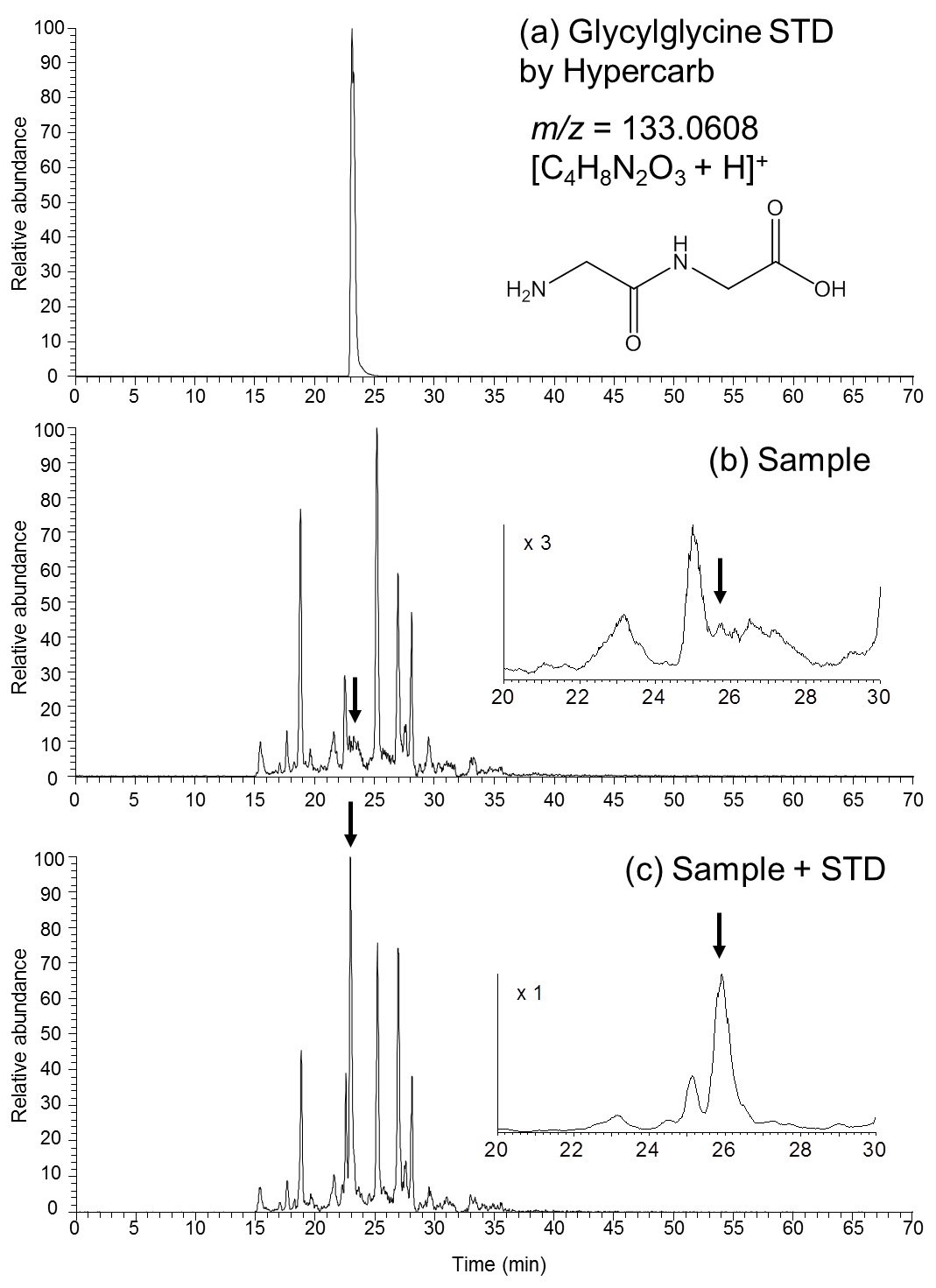


Supplementary Figure 18B. Identification of glycylglycine in the organic residues. Mass chromatograms of (a) the glycylglycine standard, (b) the analyte sample and (c) the co-injected mixture of glycylglycine standard and analyte sample at the *m/z* of 133.0608. A Hypercarb^TM^ separation column was used for the HPLC/HRMS analysis. The solid arrow indicates glycylglycine. The inset shows an enlarged spectrum at 20 to 30 min.


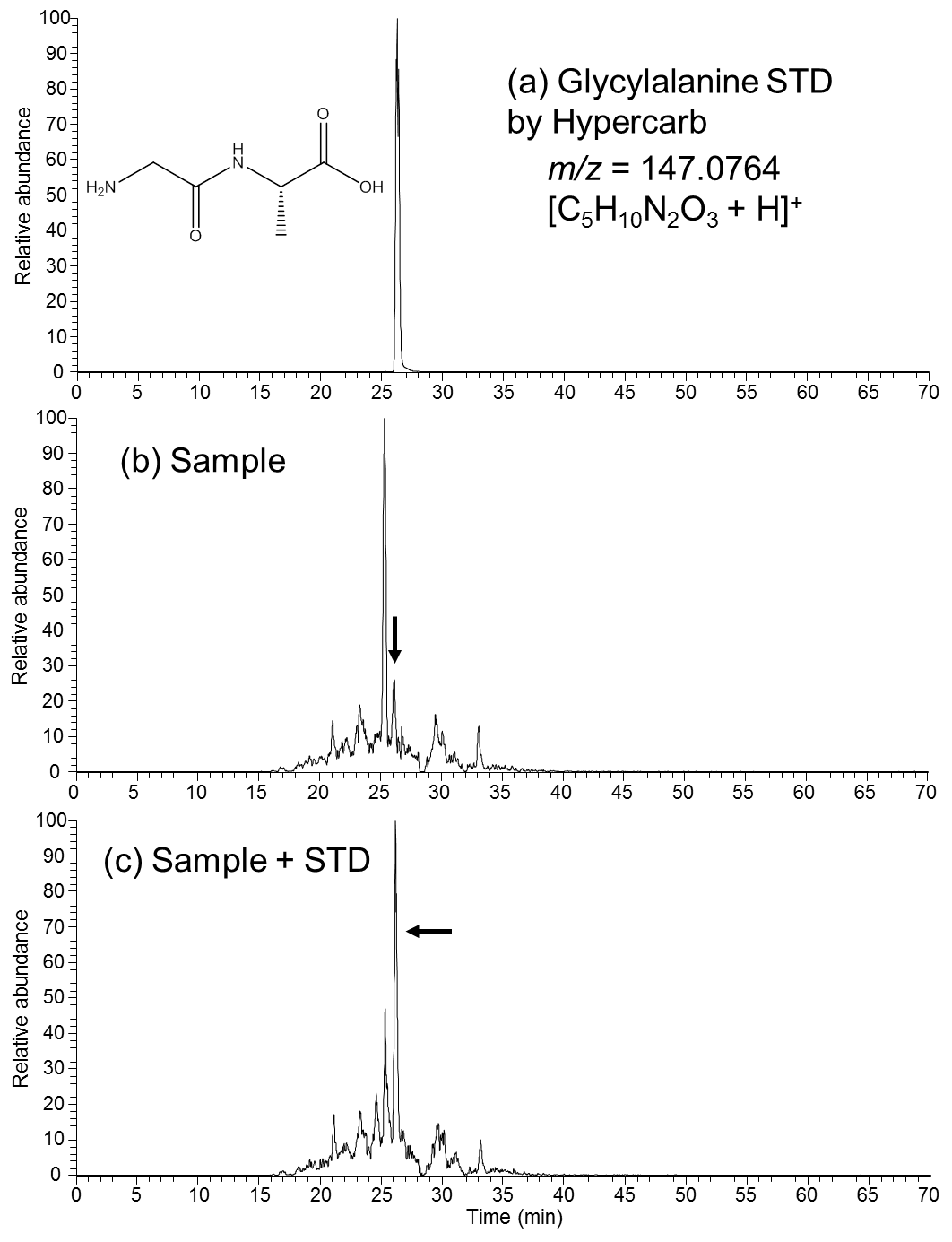


Supplementary Figure 19. Identification of glycylalanine in the organic residues. Mass chromatograms of (a) the glycylalanine standard, (b) the analyte sample and (c) the co-injected mixture of glycylalanine standard and analyte sample at the *m/z* of 147.0764. A Hypercarb^TM^ separation column was used for the HPLC/HRMS analysis. The solid arrow indicates glycylalanine.


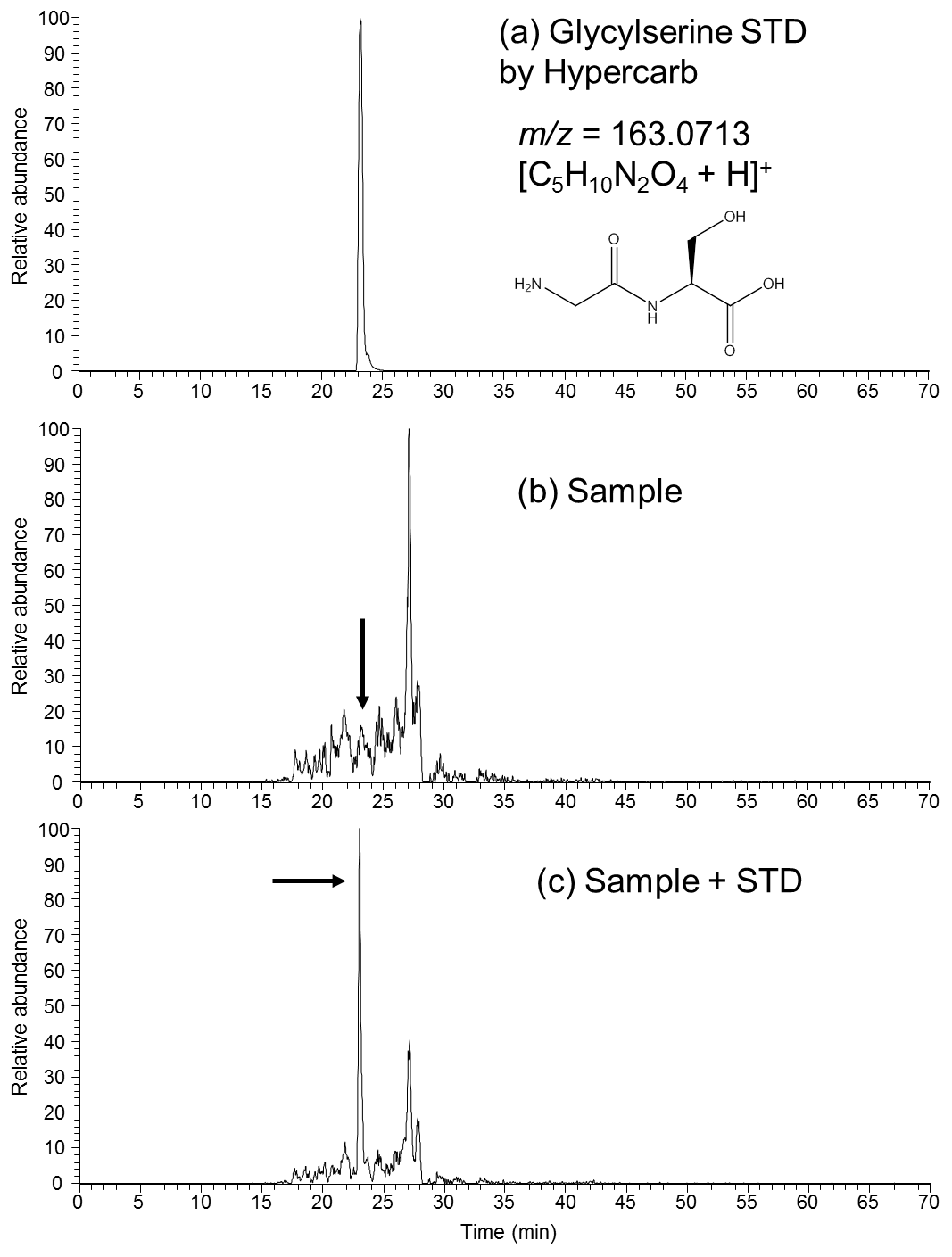


Supplementary Figure 20. Identification of glycylserine in the organic residues. Mass chromatograms of (a) the glycylserine standard, (b) the analyte sample and (c) the co-injected mixture of glycylserine standard and analyte sample at the *m/z* of 163.0713. A Hypercarb^TM^ separation column was used for the HPLC/HRMS analysis. The solid arrow indicates glycylserine.

Supplementary Figure 21. Identification of deuterated uracil in the isotopically labelled organic residues. Mass spectra of (a) uracil standard reagent and (b) the D-substituted sample at the *m*/*z* from 113.0 to 114.1. The inset shows an enlarged one at the *m*/*z* from 114.026 to 114.046. ^13^C-uracil and d_1_-uracil represent uracil isotopologues whose one carbon is replaced with ^13^C and whose one hydrogen atom is replaced with deuterium, respectively. The numbers on the detected peaks indicate the measured values of *m*/*z* for each peak.

Supplementary Figure 22. Identification of deuterated cytosine in the isotopically labelled organic residues. Mass spectra of (a) cytosine standard reagent and (b) the D-substituted sample at the *m*/*z* from 112.0 to 113.2. The inset shows an enlarged one at the *m*/*z* from 113.050 to 113.070. ^13^C-cytosine and d_1_-cytosine represent cytosine isotopologues whose one carbon is replaced with ^13^C and whose one hydrogen atom is replaced with deuterium, respectively. The numbers on the detected peaks indicate the measured values of *m*/*z* for each peak.

Supplementary Figure 23. Identification of deuterated-thymine in the isotopically labelled organic residues. Mass spectra of (a) thymine standard reagent and (b) the D-substituted sample at the *m*/*z* from 127.0 to 129.2. The insets show enlarged ones at the *m*/*z* from 128.02 to 128.10 and from 129.00 to 129.10. ^13^C-thymine, ^18^O-thymine, d_1_-thymine, and d_2_-thymine represent thymine isotopologues whose carbon is replaced with ^13^C, whose oxygen is replaced with ^18^O, whose hydrogen is replaced with deuterium, and whose two hydrogen atoms are replaced with two deuterium atoms, respectively. The numbers on the detected peaks indicate the measured values of *m*/*z* for each peak.


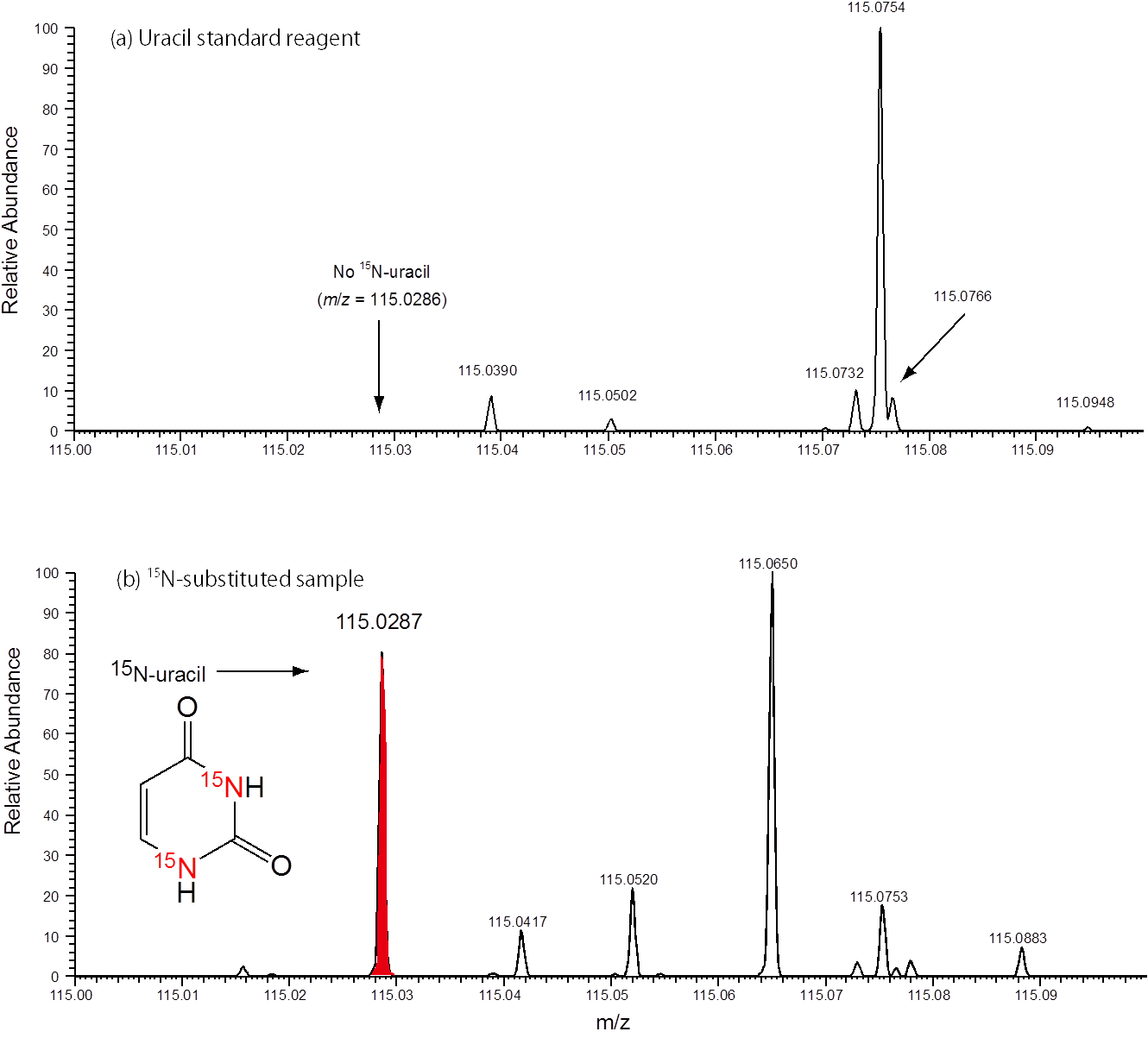


Supplementary Figure 24. Identification of ^15^N-substituted uracil in the isotopically labelled organic residues. Mass spectra of (a) uracil standard reagent and (b) of the ^15^N-substituted sample at the *m*/*z* from 115.0 to 115.1 at the retention time of ~8.5 minutes on each mass chromatogram (cf. Figure 2). A C18 separation column was used in this analysis. ^15^N-uracil represents the uracil isotopologue whose all nitrogen atoms are replaced with ^15^N (C_4_H_4_^15^N_2_O_2_: the *m*/*z* of the protonated ion is 115.0286). The numbers on the detected peaks indicate the measured values of *m*/*z* for each peak. Fully ^15^N-substituted uracil was not observed in the standard reagent, while it was observed in the ^15^N-substituted sample.


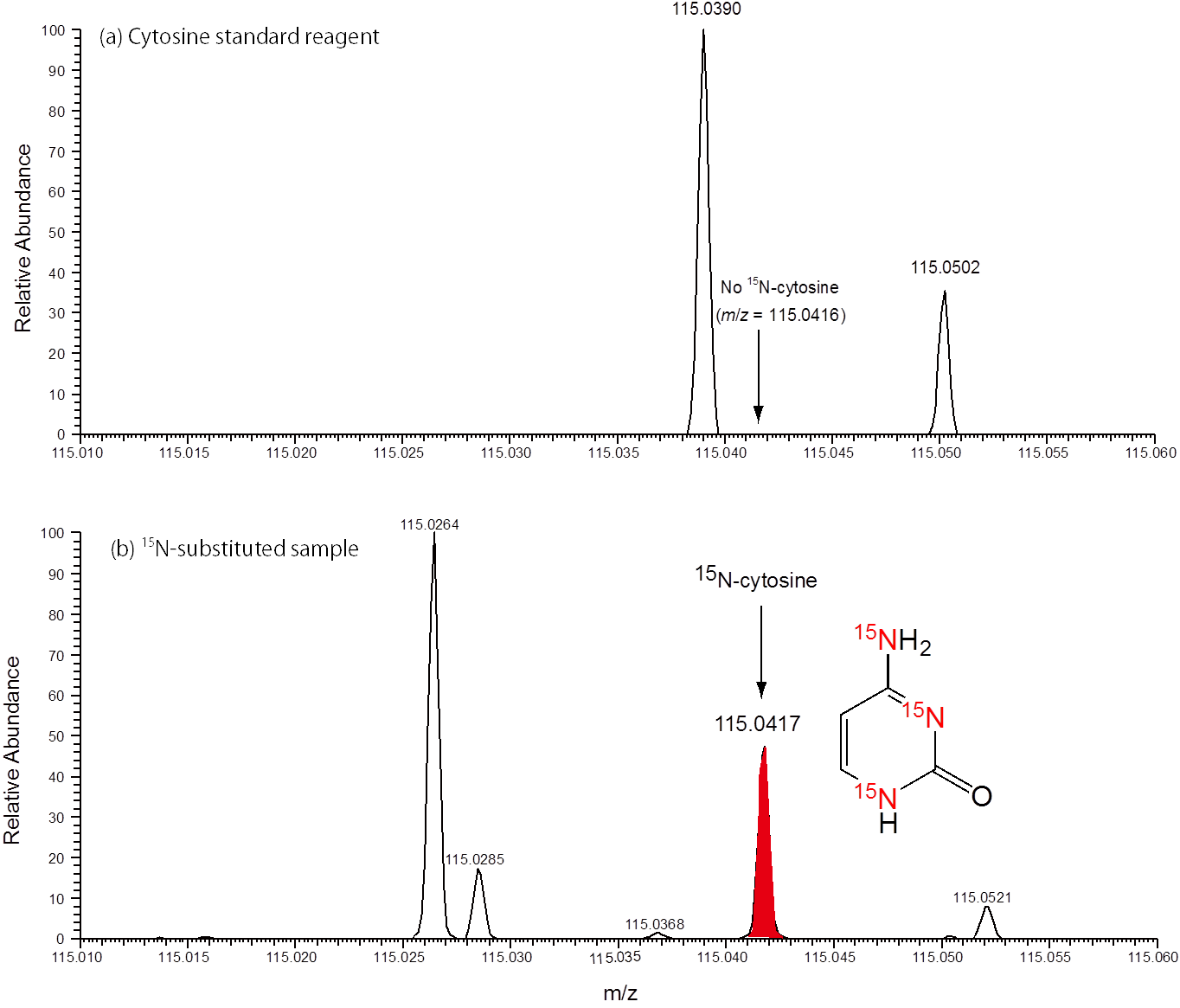


Supplementary Figure 25. Identification of ^15^N-substituted cytosine in the isotopically labelled organic residues. Mass spectra of (a) cytosine standard reagent and (b) of the ^15^N-substituted sample at the *m*/*z* from 115.01 to 115.06 at the retention time of ~5 minutes on each mass chromatogram (cf. Supplementary Figure 3A). A C18 separation column was used in this analysis. ^15^N-cytosine represents the cytosine isotopologue whose all nitrogen atoms are replaced with ^15^N (C_4_H_5_^15^N_3_O: the *m*/*z* of the protonated ion is 115.0416). The numbers on the detected peaks indicate the measured values of *m*/*z* for each peak. Fully ^15^N-substituted cytosine was not observed in the standard reagent, while it was observed in the ^15^N-substituted sample.


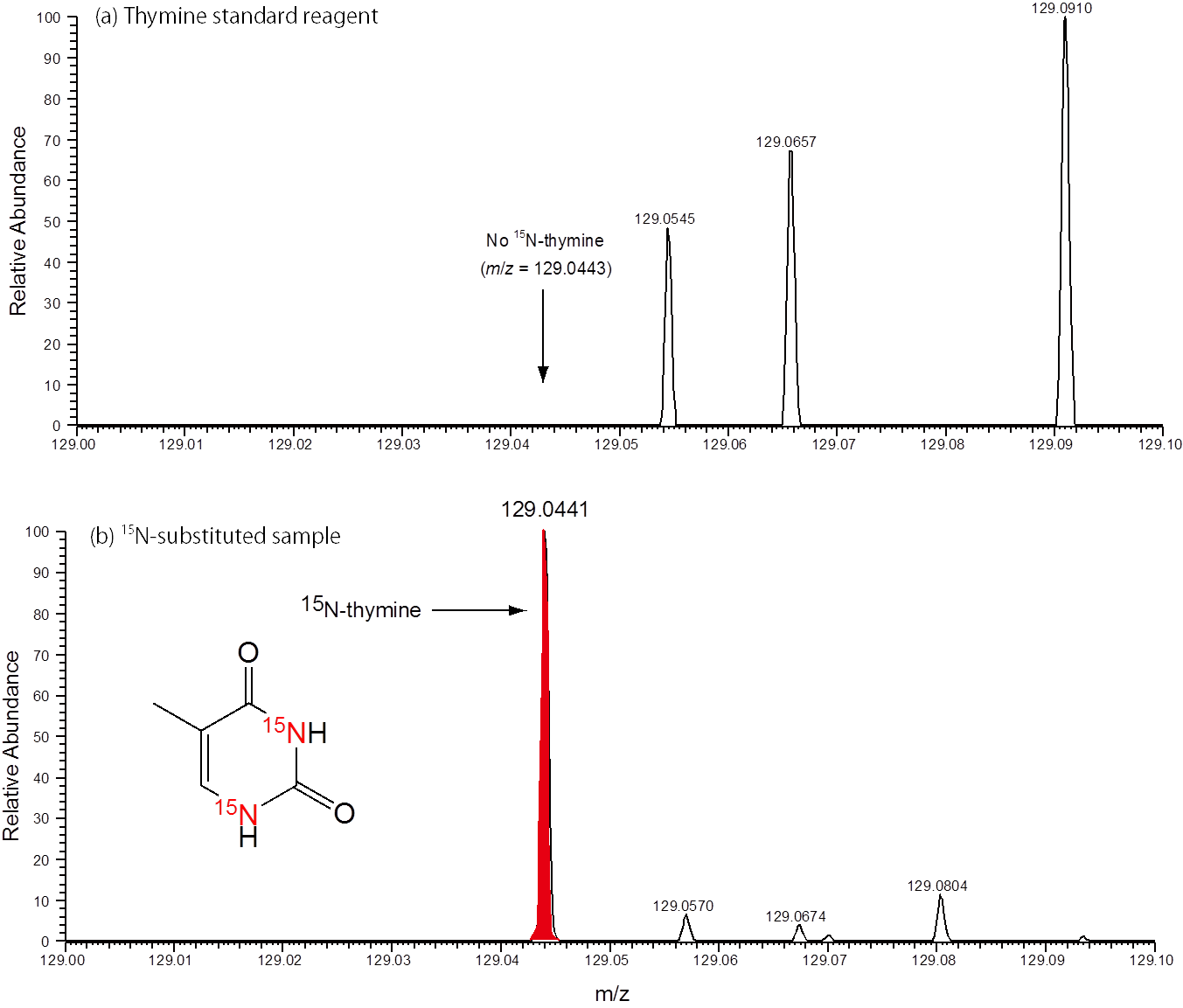


Supplementary Figure 26. Identification of ^15^N-substituted thymine in the isotopically labelled organic residues. Mass spectra of (a) thymine standard reagent and (b) of the ^15^N-substituted sample at the *m*/*z* from 129.0 to 129.1 at the retention time of ~17 minutes on each mass chromatogram (cf. Supplementary Figure 4A). A C18 separation column was used in this analysis. ^15^N-thymine represents the thymine isotopologue whose all nitrogen atoms are replaced with ^15^N (C_5_H_6_^15^N_2_O_2_: the *m*/*z* of the protonated ion is 129.0443). The numbers on the detected peaks indicate the measured values of *m*/*z* for each peak. Fully ^15^N-substituted thymine was not observed in the standard reagent, while it was observed in the ^15^N-substituted sample.


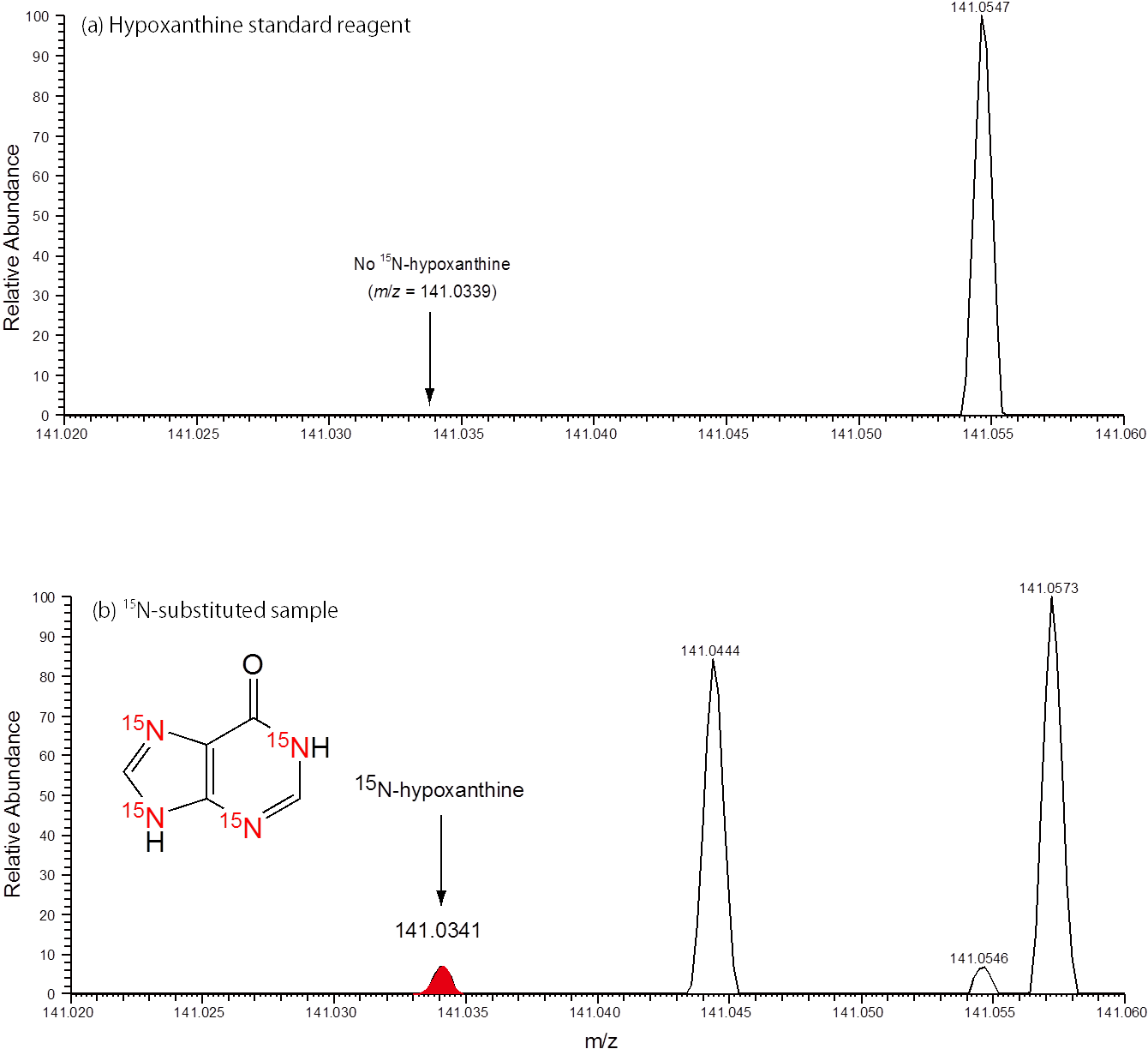


Supplementary Figure 27. Identification of ^15^N-substituted hypoxanthine in the isotopically labelled organic residues. Mass spectra of (a) hypoxanthine standard reagent and (b) of the ^15^N-substituted sample at the *m*/*z* of 141.02 to 141.06 at the retention time of ~11.5 minutes on each mass chromatogram (cf. Figure 3). A C18 separation column was used in this analysis. ^15^N-hypoxanthine represents the hypoxanthine isotopologue whose all nitrogen atoms are replaced with ^15^N (C_5_H_4_^15^N_4_O: the *m*/*z* of the protonated ion is 141.0339). The numbers on the detected peaks indicate the measured values of *m*/*z* for each peak. Fully ^15^N-substituted hypoxanthine was not observed in the standard reagent, while it was observed in the ^15^N-substituted sample.


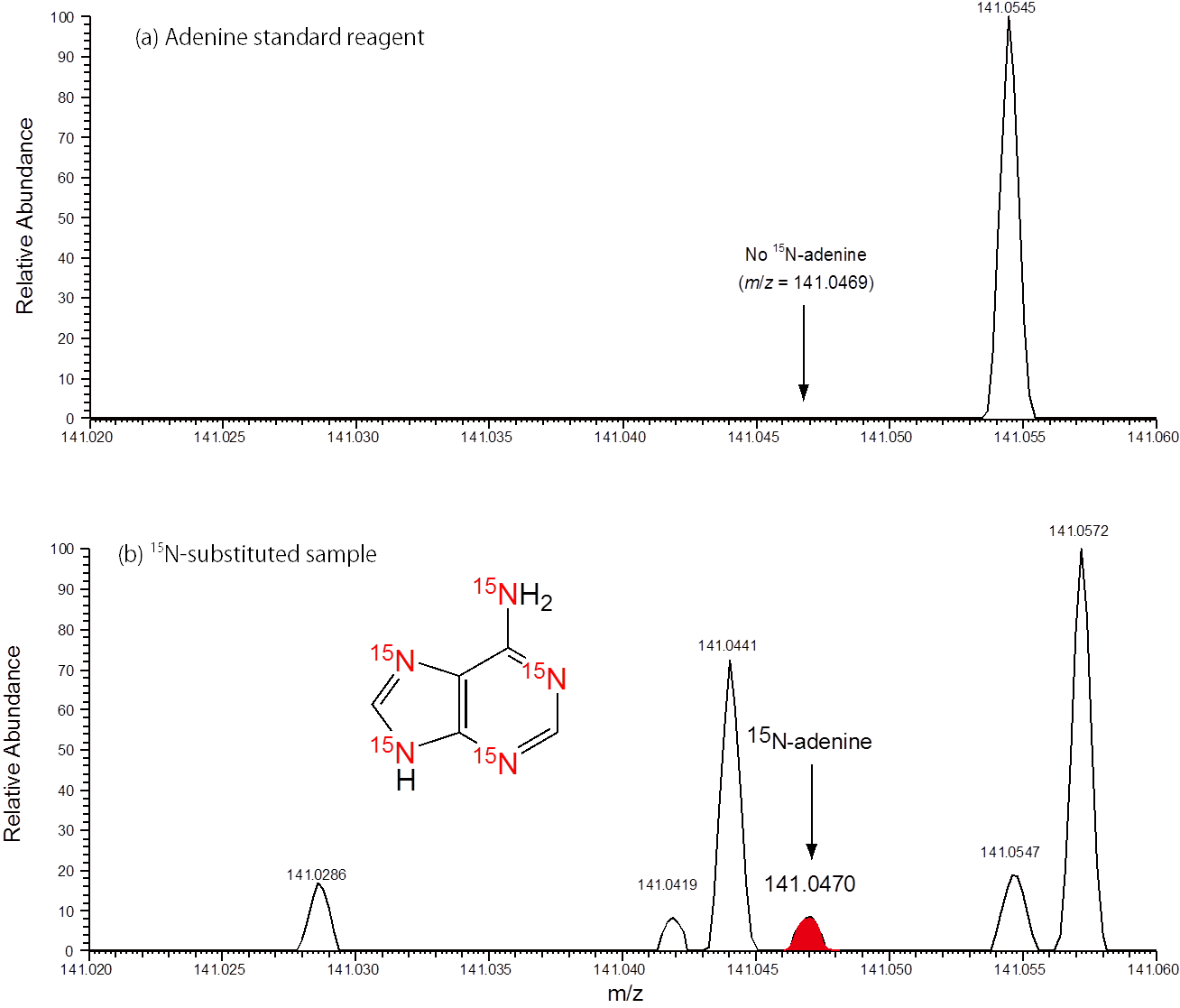


Supplementary Figure 28. Identification of ^15^N-substituted adenine in the isotopically labelled organic residues. Mass spectra of (a) adenine standard reagent and (b) of the ^15^N-substituted sample at the *m*/*z* of 141.02 to 141.06 at the retention time of ~8 minutes on each mass chromatogram (cf. Supplementary Figure 5). A C18 separation column was used in this analysis. ^15^N-adenine represents the adenine isotopologue whose all nitrogen atoms are replaced with ^15^N (C_5_H_5_^15^N_5_: the *m*/*z* of the protonated ion is 141.0469). The numbers on the detected peaks indicate the measured values of *m*/*z* for each peak. Fully ^15^N-substituted adenine was not observed in the standard reagent, while it was observed in the ^15^N-substituted sample.


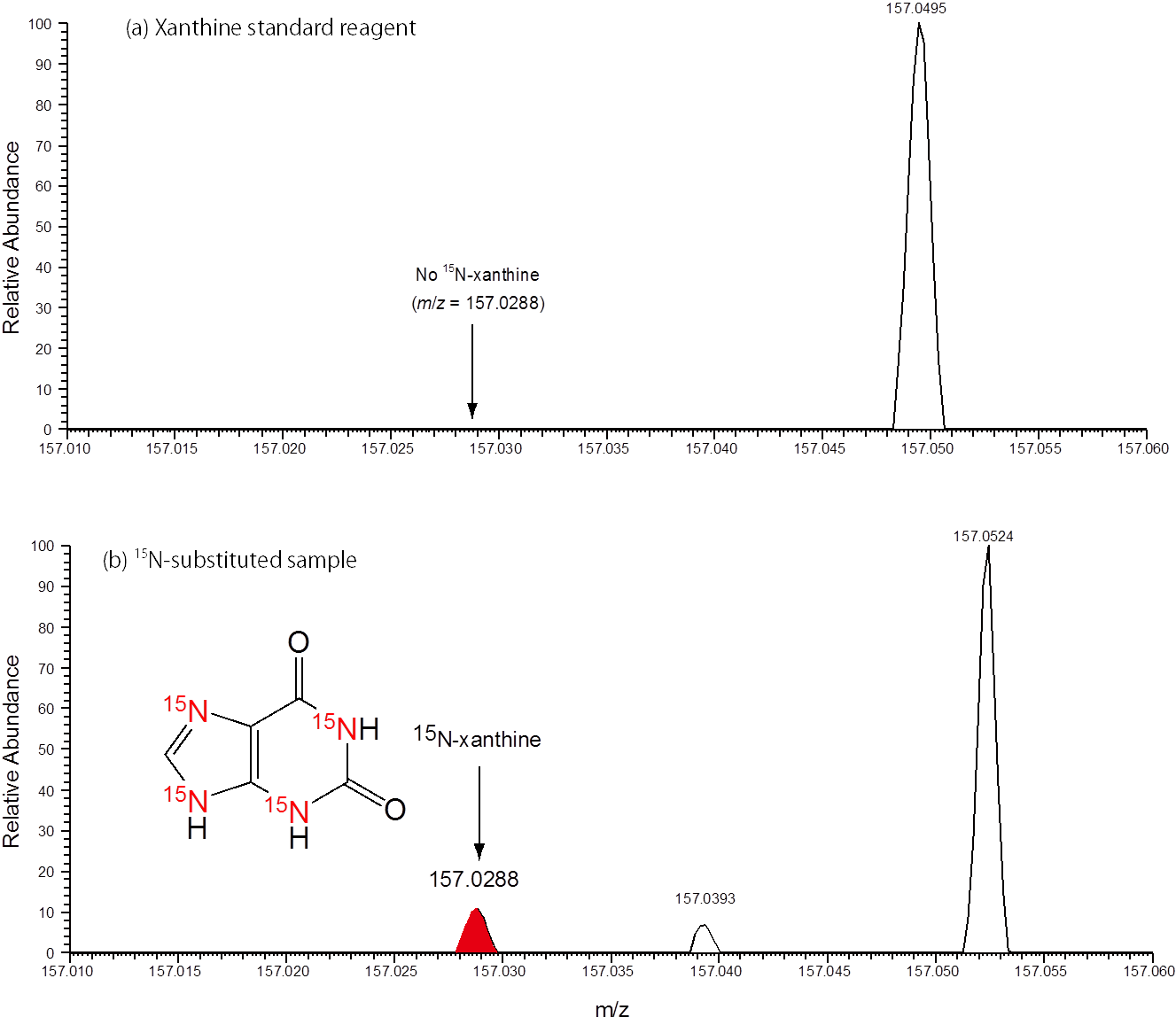


Supplementary Figure 29. Identification of ^15^N-substituted xanthine in the isotopically labelled organic residues. Mass spectra of (a) xanthine standard reagent and (b) of the ^15^N-substituted sample at the *m*/*z* of 157.01 to 157.06 at the retention time of ~14 minutes on each mass chromatogram (cf. Supplementary Figure 6). A C18 separation column was used in this analysis. ^15^N-xanthine represents the xanthine isotopologue whose all nitrogen atoms are replaced with ^15^N (C_5_H_4_^15^N_4_O_2_: the *m*/*z* of the protonated ion is 157.0288). The numbers on the detected peaks indicate the measured values of *m*/*z* for each peak. Fully ^15^N-substituted xanthine was not observed in the standard reagent, while it was observed in the ^15^N-substituted sample.


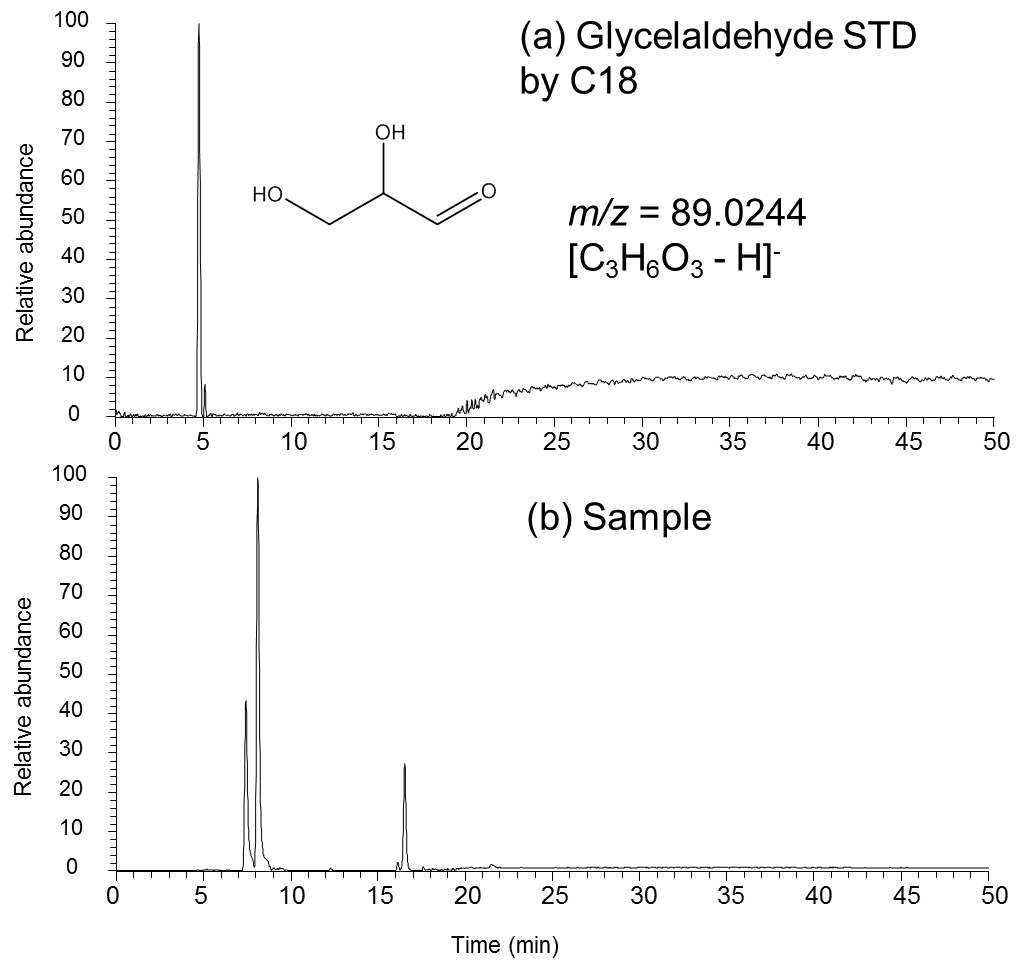


Supplementary Figure 30. Search for glycelaldehyde in the organic residues. Mass chromatograms of (a) the glyceraldehyde standard and (b) the analyte sample at a mass-to-charge ratio (*m*/*z*) of 89.0244. A C18 separation column was used for the analysis performed by the HPLC/HRMS analysis. Glyceraldehyde was not positively identified on the sample spectrum.


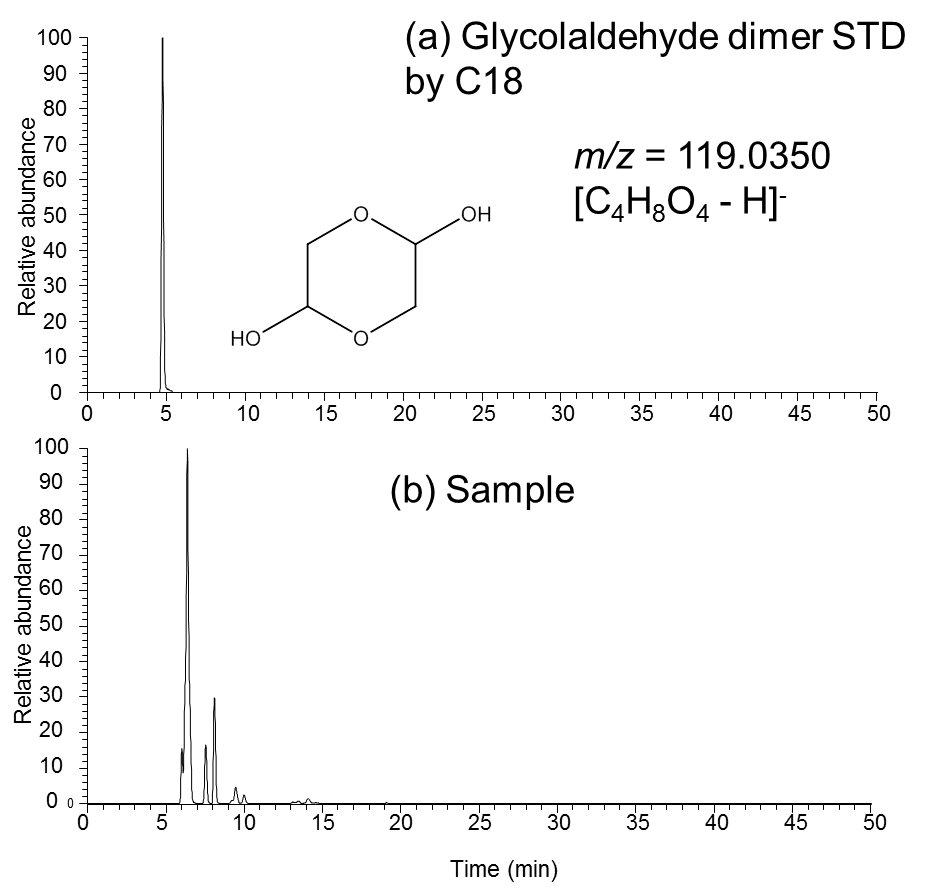


Supplementary Figure 31. Search for glycolaldehyde dimer in the organic residues. Mass chromatograms of (a) the glycolaldehyde dimer standard and (b) the analyte sample at a mass-to-charge ratio (*m*/*z*) of 119.0350. A C18 separation column was used for the analysis by the HPLC/HRMS analysis. Glycolaldehyde dimer was not positively identified on the sample spectrum.


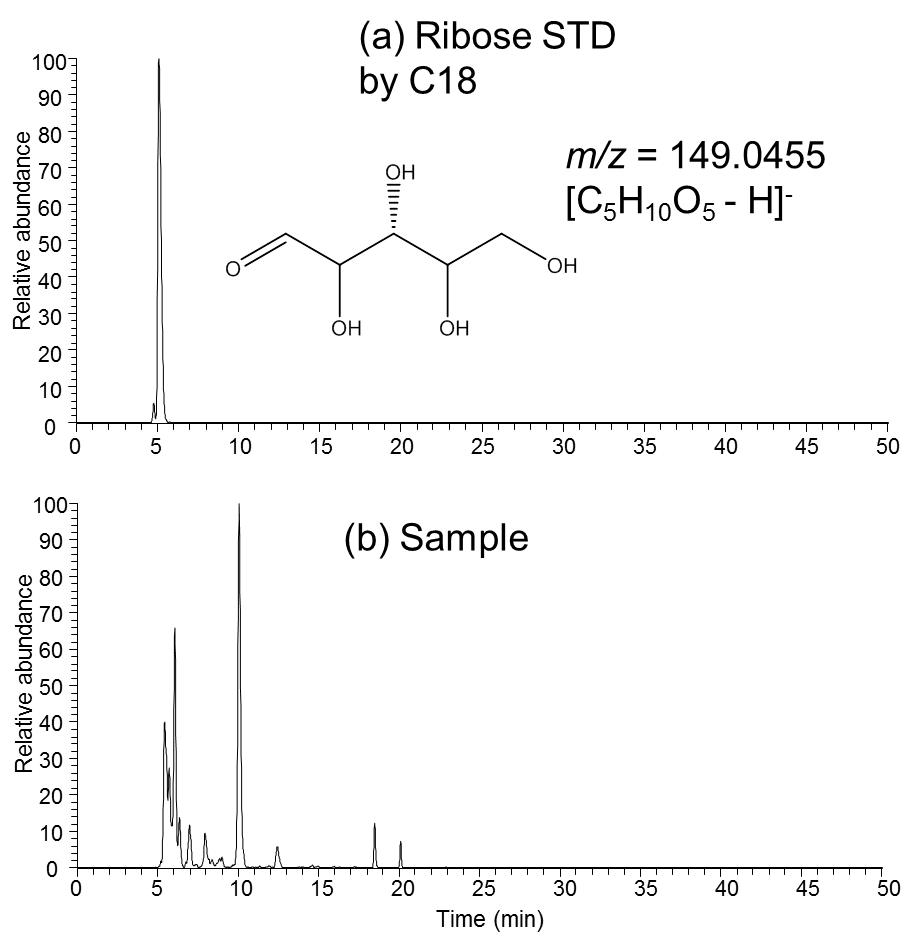


Supplementary Figure 32. Search for ribose in the organic residues. Mass chromatograms of (a) the ribose standard and (b) the analyte sample at a mass-to-charge ratio (*m/z*) of 149.0455. A C18 separation column was used for the analysis performed by the HPLC/HRMS analysis. Due to the poor separation between the peaks due to ribose and those due to ribose isomers under the analytical conditions utilised, an exact quantification of ribose was not possible. Instead, the upper limit of the yield was provided to be 12 ppm, a value that was estimated by assuming that the peak observed at about the 5-min mark in the sample mass chromatogram is derived from ribose.


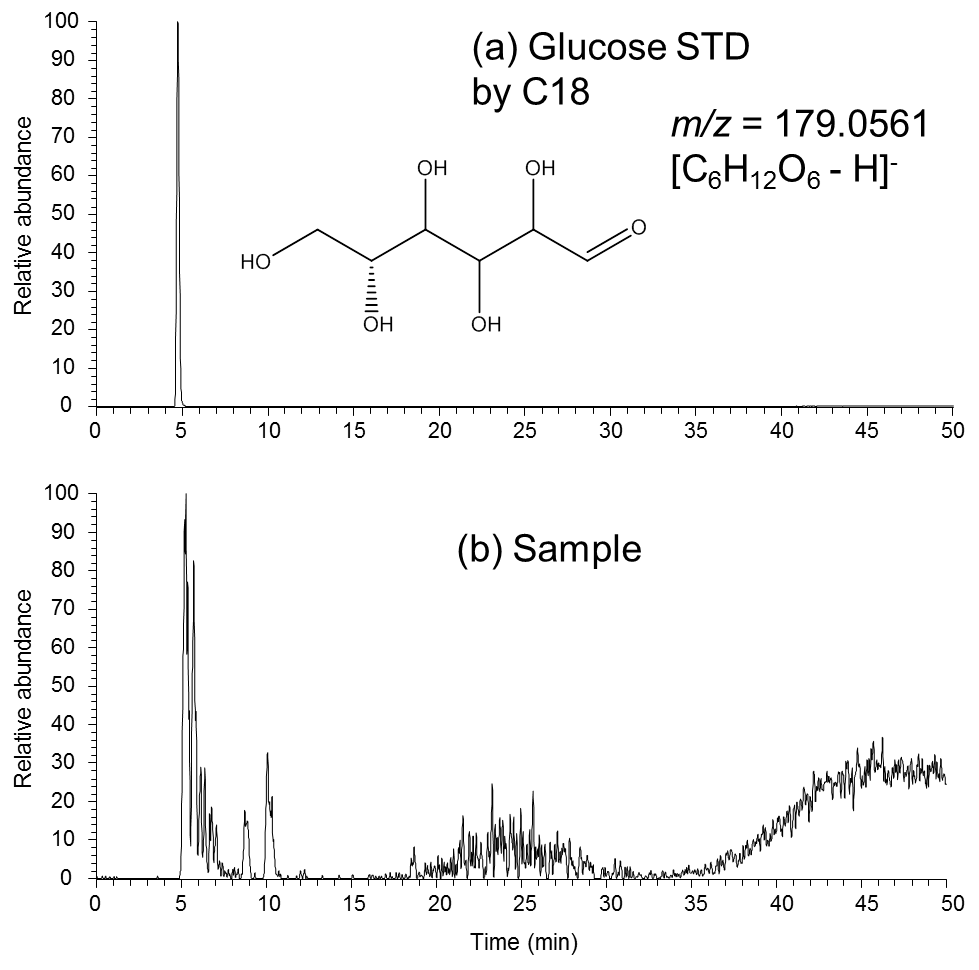


Supplementary Figure 33. Search for glucose in the organic residues. Mass chromatograms of (a) the glucose standard and (b) the analyte sample at a mass-to-charge ratio (*m/z*) of 179.0561. A C18 separation column was used for the analysis by the HPLC/HRMS analysis. Due to the poor separation between the peaks due to glucose and those due to glucose isomers under the analytical conditions utilised, an exact quantification of glucose was not possible. Instead, the upper limit of the yield was provided to be 1 ppm, a value that was estimated by assuming that the peak observed at about the 5-min mark in the sample mass chromatogram is derived from glucose.

Supplementary Figure 34. The recovery (%) of the cation-exchange column chromatography showing representative aliphatic (Val, Valine), N-heterocyclic (Pro, Proline; Hyp, Hydroxyproline), aromatic (Phe, Phenylalanine; Tyr, Tyrosine) N-containing molecules^14^. The recovery (%) of most abundant nucleobase, Cytosine (Cyt), was also compiled for the present study. 2-Aminoadipic acid (2-AAA) is one of internal standards for laboratory wet chemical treatment. As an individual verification after the ref. 39, we note that the recovery (%) of N-containing molecules showed better than 95.0 ± 3.5% (ave.± SD, *n* = 3) including the N-heterocyclic derivatives of -Proline, -Histidine, -Phenylalanine, -Tryptophan as model compounds^14^.

Supplementary Figure 35. Examples of calibration curves for nucleobases measured by HPLC/HRMS. (a) Uracil, (b) hypoxanthine. The solid red line is the linear fit to the plots in each data.

**Supplementary Tables**

| Supplementary Table 1. Quantification results for dipeptides and amino acids in the organic residue. | | | | |  |
| --- | --- | --- | --- | --- | --- |
| Name of molecule | Molecular formula | Molecular structure | Mass to charge ratio (*m*/*z*) of the protonated ion | Yield (ppm)* by a C18 column | Yield (ppm)* by a Hypercarb^TM^ column |
| Glycylglycine | C_4_H_8_N_2_O_3_ | (S1) | 133.0608 | <0.4 | 12 |
| Glycylalanine | C_5_H_10_N_2_O_3_ | (S2) | 147.0764 | - | 4 |
| Glycylserine | C_5_H_10_N_2_O_4_ | (S3) | 163.0713 | - | 4 |
| Glycine | C_2_H_5_NO_2_ | (S4) | 76.0393 | - | 270 |
| α-Alanine | C_3_H_7_NO_2_ | (S5) | 90.055 | - | 304 |
| β-Alanine | C_3_H_7_NO_2_ | (S6) | 90.055 | - | 323 |
| Sarcosine | C_3_H_7_NO_2_ | (S7) | 90.055 | - | 212 |
| *Relative weight with relevance to the total deposited gas in part per million (1 ppm = 0.0001%) normalised with each carbon abundance | | | | | |

| Supplementary Table 2. Upper limit for sugars in the present organic residue. | | | |
| --- | --- | --- | --- |
| Name of molecule | Molecular formula | Mass to charge ratio (*m*/*z*) of the protonated ion | Upper limit of the yield (ppm)* |
| Glycelaldehyde | C_3_H_6_O_3_ | 89.0244 | - |
| Glycolaldehyde dimer | C_4_H_8_O_4_ | 119.0350 | - |
| Ribose | C_5_H_10_O_5_ | 149.0455 | 12 |
| Glucose | C_6_H_12_O_6_ | 179.0561 | 1 |
| *Relative weight with relevance to the total deposited gas in part per million (1 ppm = 0.0001%) normalised with each carbon abundance.  Note: The determination of sugar-related compounds was conducted without the cation-exchange chromatography. | | | |

**Supplementary References**

1. Hama, T. & Watanabe, N. Surface processes on interstellar amorphous solid water: adsorption, diffusion, tunneling reactions, and nuclear-spin conversion. *Chem. Rev.* **113**, 8783–8839 (2013).
2. Wang, T. & Bowie, J. H. Can cytosine, thymine and uracil be formed in interstellar regions? A theoretical study. *Org. Biomol. Chem.* **10**, 652–662 (2012).
3. Snyder, L. E. & Buhl, D. Interstellar isocyanic acid. *Astrophys. J.* **177**, 619–623 (1972).
4. Hollis, J. M. et al. Green bank telescope detection of new interstellar aldehydes: propenal and propanal. *Astrophys. J. Lett.* **610**, L21–L24 (2004).
5. Fedoseev, G. et al. Simultaneous hydrogenation and UV-photolysis experiments of NO in CO-rich interstellar ice analogues; linking HNCO, OCN^-^, NH_2_CHO, and NH_2_OH. *Mon. Not. R. Astron. Soc.* **460**, 4297–4309 (2016).
6. Saladino, R. et al. Meteorite-catalyzed syntheses of nucleosides and of other prebiotic compounds from formamide under proton irradiation. *Proc. Nat. Acad. Sci. USA* **112**, E2746–E2755 (2015).
7. Barks, H. L. et al. Guanine, adenine, and hypoxanthine production in UV-irradiated formamide solutions: relaxation of the requirements for prebiotic purine nucleobase formation. *ChemBioChem* **11**, 1240–1243 (2010).
8. Majumdar, L., Gorai, P., Das, A. & Chakrabarti, S. K. Potential formation of three pyrimidine bases in interstellar regions. *Astrophys. Space Sci.* **360**, 64 (2015).
9. Ferus, M. et al. High-energy chemistry of formamide: a unified mechanism of nucleobase formation. *Proc. Nat. Acad. Sci. USA* **112**, 657–662 (2015).
10. Ferus, M. et al. High-energy chemistry of formamide: a simpler way for nucleobase formation. *J. Phys. Chem. A* **118**, 719–736 (2014).
11. Grim, R. J. A. et al. Infrared spectroscopy of astrophysical ices: new insights in the photochemistry. *Astron. Astrophys. Suppl. Ser.* **78**, 161–186 (1989).
12. Jones, B. M., Bennett, C. J. & Kaiser, R. I. Mechanistical studies on the production of formamide (H_2_NCHO) within interstellar ice analogs. *Astrophys. J.* **734**, 78 (2011).
13. Tachibana*,* S. et al. Liquid-like behavior of UV-irradiated interstellar ice analog at low temperatures. *Sci. Adv.* **3**, eaao2538 (2017).
14. Takano, Y., Furota, S., Ogawa, O. N. and Ohkouchi N. Analytical development of underivatized amino acids and peptide molecules. *Abstract for Annual Meetings of the Meteoritical Society* (2019).
